# Supplementary material for: Added value of electrical impedance spectroscopy in adjunction of colposcopy: a prospective cohort study
Source: BMJ Open. 2023 Oct 29;13(10):e074921. doi: 10.1136/bmjopen-2023-074921 (PMC10619076; doi:10.1136/bmjopen-2023-074921)
Supplement: Supplementary data [file bmjopen-2023-074921supp001.pdf]

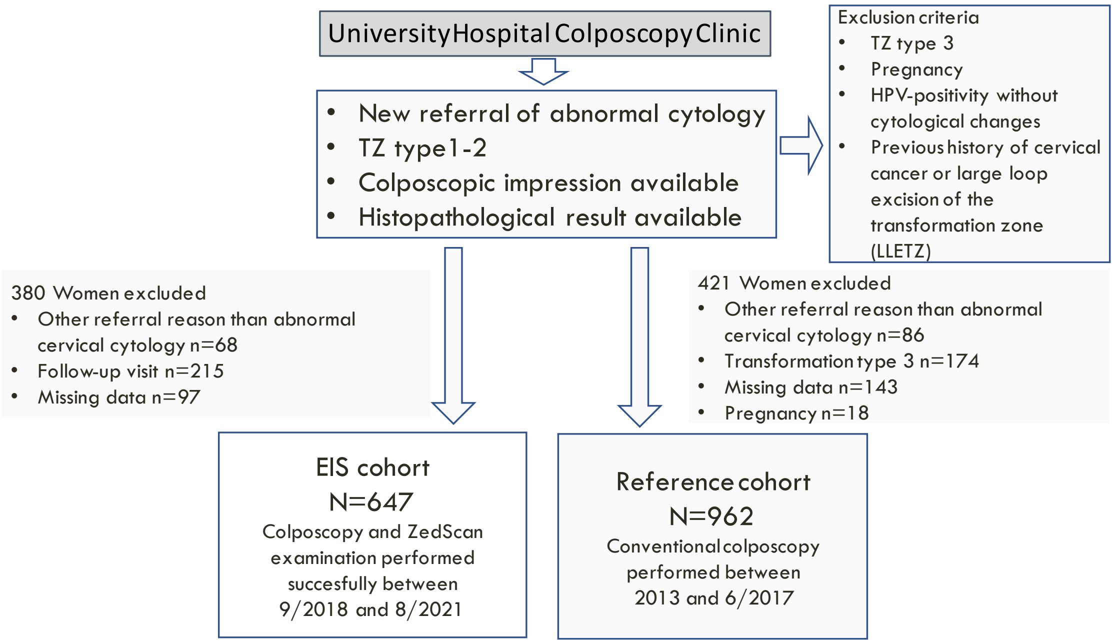

Figure S1. Flow-chart of the study comparing the performance of colposcopy by referral cervical cytology in two cohorts with and without electrical impedance spectroscopy as an adjunctive technology.
