# Supplementary material for: Added value of electrical impedance spectroscopy in adjunction of colposcopy: a prospective cohort study
Source: BMJ Open. 2023 Oct 29;13(10):e074921. doi: 10.1136/bmjopen-2023-074921 (PMC10619076; doi:10.1136/bmjopen-2023-074921)
Supplement: Supplementary data [file bmjopen-2023-074921supp002.pdf]

 BMJ Publishing Group Limited (BMJ) disclaims all liability and responsibility arising from any reliance placed on this supplemental material which has been supplied by the author(s)

Table S1 Sensitivity, specificity, negative and positive predictive value of the electrical impedance spectroscopy (EIS) cohort and the reference cohort for the detection of CIN2+ lesions by cervical cytology, TZ type and age group.

	EIS cohort (n=647)									Reference cohort (n=962)								
	CIN2+/n	CIN2%	Colpo+ZS CIN2+[1]	Sensitivity	<CIN2/n	Colpo +ZS <CIN2[3]	Specificity	PPV	NPV	CIN2+/n	CIN2%	Colpo CIN2+[2]	Sensitivity	<CIN2/n	Colpo <CIN2[4]	Specificity	PPV	NPV
ALL	222/647	34.3	209	94(90-97)	425/647	144	34(29-39)	43(38-47)	92(86-96)	391/962	40.6	267	68(63-73)	571/962	480	84(81-87)	75(70-79)	80(76-83)
ASC-US	16/94	17.0	15	94(70-100)	78/94	37	47(36-59)	27(16-40)	97(86-100)	9/99	9.1	5	56(21-86)	90/99	87	97(91-99)	63(25-92)	96(89-99)
LSIL	39/236	16.5	30	77(61-89)	197/236	82	42(35-49)	21(14-28)	90(82-95)	72/381	18.9	31	43(31-55)	309/381	285	92(89-95)	56(42-70)	87(83-91)
ASC-H	85/192	44.3	84	99(94-100)	107/192	12	11(6-19)	47(39-55)	92(64-100)	138/237	58.2	87	63(54-71)	99/237	64	65(54-74)	71(62-79)	56(46-65)
HSIL	77/94	81.9	77	100(95-100)	17/94	1	6(0-29)	83(74-90)	100(3-100)	154/200	77.0	133	86(80-91)	46/200	21	46(31-61)	84(78-90)	50(34-66)
AGC-NOS	3/28	10.7	2	67(9-99)	25/28	11	44(24-65)	13(2-38)	92(62-100)	5/28	17.9	3	60(15-95)	23/28	22	96(78-100)	75(19-99)	92(73-99)
AGC-FN	2/3	66.7	1	50(1-99)	1/3	1	100(3-100)	100(3-100)	50(1-99)	13/17	76.5	8	62(32-86)	4/17	1	25(1-81)	73(39-94)	17(0-64)
TZ1	156/446	35.0	146	94(89-97)	290/446	90	31(26-37)	42(37-48)	90(82-95)	279/620	45.0	187	67(61-73)	341/620	287	84(80-88)	78(72-83)	76(71-80)
TZ2	66/201	32.8	63	95(87-99)	135/201	54	40(32-49)	44(36-52)	95(85-99)	112/342	32.7	80	71(62-80)	230/342	193	84(79-88)	68(59-77)	86(81-90)
<30 y	60/175	34.3	56	93(84-98)	115/175	40	35(26-44)	43(34-52)	91(78-98)	134/295	45.4	96	72(63-79)	161/295	124	77(70-83)	72(64-80)	77(69-83)
30-44 y	131/366	35.8	124	95(89-98)	235/366	78	33(27-40)	44(38-50)	92(84-97)	211/495	42.6	144	68(62-75)	284/495	244	86(81-90)	78(72-84)	79(74-83)
≥ 45 y	31/106	29.2	29	94(79-99)	75/106	26	35(24-47)	37(27-49)	93(77-99)	46/172	26.7	27	59(43-73)	126/172	112	89(82-94)	66(49-80)	86(78-91)
HG cytology	164/289	56.7	162	99(96-100)	125/289	14	11(6-18)	59(53-65)	88(62-98)	305/454	67.2	228	75(70-80)	149/454	86	58(49-66)	78(73-83)	53(45-61)
LG cytology	58/358	16.2	47	81(69-90)	300/358	130	43(38-49)	22(16-28)	92(87-96)	86/508	16.9	39	45(35-57)	422/508	394	93(91-96)	58(46-70)	89(86-92)
ASC-H, HSIL	162/286	56.6	161	99(97-100)	124/286	13	10(6-17)	59(53-65)	93(66-100)	292/437	66.8	220	75(70-80)	145/437	85	59(50-67)	79(73-83)	54(46-62)
ASC-US,LSIL	55/330	16.7	45	82(69-91)	275/330	119	43(37-49)	22(17-29)	92(86-96)	81/480	16.9	36	44(33-56)	399/480	372	93(90-96)	57(44-70)	89(86-92)
Glandular	5/31	16.1	3	60(15-95)	26/31	12	46(27-67)	18(4-43)	86(57-98)	18/45	40.0	11	61(36-83)	27/45	23	85(66-96)	73(45-92)	77(58-90)
1 biopsy	11/165	6.7	7	64(31-89)	154/165	78	51(43-59)	8(4-17)	95(88-99)	14/109	12.8	5	36(13-65)	95/109	94	99(94-100)	83(36-100)	91(84-96)
2 biopsies	78/263	29.7	70	90(81-96)	185/263	43	23(17-30)	33(27-40)	84(71-93)	112/420	26.7	66	59(49-68)	308/420	276	90(86-93)	67(57-77)	86(81-89)
≥3 biopsies	43/84	51.2	43	100	41/84	0	0	51	0	168/305	55.1	113	67(60-74)	137/305	92	67(59-75)	72(64-78)	63(54-70)
LLETZ	90/113	79.6	89	99(94-100)	23/113	1	4(0-22)	80(72-87)	50(1-99)	97/118	82.2	83	86(77-92)	21/118	8	38(18-62)	87(78-93)	36(17-59)

[1]Colposcopic impression and/or ZedScan result of CIN2+ of histologically confirmed CIN2+ cases.

[2]Colposcopic impression of CIN2+ of histologically confirmed CIN2+ cases.

[3]Colposcopic impression and ZedScan result less than CIN2 of histologically confirmed cases <CIN2.

[4]Colposcopic impression less than CIN2 of histologically confirmed cases <CIN2

AGC-FN: atypical glandular cells that favour neoplasia; AGC-NOS: atypical glandular cells not otherwise specified; ASC-H: atypical squamous cells that cannot exclude HSIL; ASC-US: atypical squamous cells of undetermined significance CIN: cervical intraepithelial neoplasia; HG: high grade; HSIL: high-grade squamous intraepithelial lesion; LG: low grade; LLETZ: large loop excision of the transformation zone; LSIL: low-grade squamous intraepithelial lesion; NPV: negative predictive value; PPV: positive predictive value; TZ: transformation zone
