# Supplementary material for: Added value of electrical impedance spectroscopy in adjunction of colposcopy: a prospective cohort study
Source: BMJ Open. 2023 Oct 29;13(10):e074921. doi: 10.1136/bmjopen-2023-074921 (PMC10619076; doi:10.1136/bmjopen-2023-074921)
Supplement: Supplementary data [file bmjopen-2023-074921supp003.pdf]

Table S2 Sensitivity, specificity, positive and negative predictive value of the electrical impedance spectroscopy (EIS) cohort and the reference cohort for the detection of CIN2+ lesions within different cervical cytology by TZ type and age group.

	EIS cohort (n=647)								Reference cohort (n=962)							
	CIN2+/n	Colpo+ZS[1] CIN2+	Sensitivity	<CIN/n	Colpo+ZS[3] <CIN2	Specificity	PPV	NPV	CIN2+/n	Colpo[2] CIN2+	Sensitivity	<CIN2/n	Colpo[4] <CIN2	Specificity	PPV	NPV
ASC-US	16/94	15	94(70-100)	78/94	37	47(36-59)	27(16-40)	97(86-100)	9/99	5	56(21-86)	90/99	87	97(91-99)	63(25-92)	96(89-99)
TZ1	11/66	10	91(59-100)	55/66	24	44(30-58)	24(12-40)	96(80-100)	7/57	3	43(10-82)	50/57	47	94(84-99)	50(12-88)	92(81-98)
TZ2	5/28	5	100(48-100)	23/28	13	57(35-77)	33(12-62)	100(75-100)	2/42	2	100(16-100)	40/42	40	100(91-100)	100(16-100)	100(91-100)
<30 y	6/28	5	83(36-100)	22/28	12	55(32-76)	33(12-62)	92(64-100)	1/43	0	0(0-98)	42/43	39	93(81-99)	0(0-71)	98(87-100)
30-44 y	10/52	10	100(69-100)	42/52	21	50(34-66)	32(17-51)	100(84-100)	7/28	4	57(18-90)	21/28	21	100(84-100)	100(40-100)	88(68-97)
≥45 y	0/14	0	0	14/14	4	29	0	100	1/28	1	100(3-100)	27/28	27	100(87-100)	100(3-100)	100(87-100)
LSIL	39/236	30	77(61-89)	197/236	82	42(35-49)	21(14-28)	90(82-95)	72/381	31	43(31-55)	309/381	285	92(89-95)	56(42-70)	87(83-91)
TZ1	26/157	20	77(56-91)	131/157	53	41(32-49)	20(13-30)	90(79-96)	53/235	23	43(30-58)	182/235	169	93(88-96)	64(46-79)	85(79-90)
TZ2	13/79	10	77(46-95)	66/79	29	44(32-57)	21(11-36)	91(75-98)	19/146	8	42(20-67)	127/146	116	91(85-96)	42(20-67)	91(85-96)
<30 y	4/39	1	25(1-81)	35/39	21	60(42-76)	7(0-32)	88(68-97)	17/79	9	53(28-77)	62/79	54	87(76-94)	53(28-77)	87(76-94)
30-44 y	28/153	24	86(67-96)	125/153	46	37(28-46)	23(16-33)	92(81-98)	46/224	19	41(27-57)	178/224	166	93(89-97)	61(42-78)	86(80-91)
≥45 y	7/44	5	71(29-96)	37/44	15	41(25-58)	19(6-38)	88(64-99)	9/78	3	33(8-70)	69/78	65	94(86-98)	43(10-82)	92(83-97)
ASC-H	85/192	84	99(94-100)	107/192	12	11(6-19)	47(39-55)	92(64-100)	138/237	87	63(54-71)	99/237	64	65(54-74)	71(62-79)	56(46-65)
TZ1	57/134	56	98(91-100)	77/134	6	8(3-16)	44(35-53)	86(42-100)	100/167	64	64(54-73)	67/167	42	63(50-74)	72(61-81)	54(42-65)
TZ2	28/58	28	100(88-100)	30/58	6	20(8-39)	54(40-68)	100(54-100)	38/70	23	61(43-76)	32/70	22	69(50-84)	70(51-84)	60(42-75)
<30 y	24/72	24	100(86-100)	48/72	5	10(4-23)	36(25-49)	100(48-100)	57/90	34	60(46-72)	33/90	18	55(36-72)	69(55-82)	44(29-60)
30-44 y	46/90	45	98(89-100)	44/90	4	9(3-22)	53(42-64)	80(28-100)	67/120	45	67(55-78)	53/120	37	70(56-82)	74(61-84)	63(49-75)
≥45 y	15/30	15	100(78-100)	15/30	3	20(4-48)	56(35-75)	100(29-100)	14/27	8	57(29-82)	13/27	9	69(39-91)	67(35-90)	60(32-84)
HSIL	77/94	77	100(95-100)	17/94	1	6(0-29)	83(74-90)	100(3-100)	154/200	133	86(80-91)	46/200	21	46(31-61)	84(78-90)	50(34-66)
TZ1	58/67	58	100	9/67	0	0	87	0	104/131	88	85(76-91)	27/131	15	56(35-75)	88(80-94)	48(30-67)
TZ2	19/27	19	100(82-100)	8/27	1	13(0-53)	73(52-88)	100(3-100)	50/69	45	90(78-97)	19/69	6	32(13-57)	78(65-88)	55(23-83)
<30 y	25/31	25	100	6/31	0	0	81	0	54/75	48	89(77-96)	21/75	10	48(26-70)	81(69-90)	63(35-85)
30-44 y	45/54	45	100(92-100)	9/54	1	11(0-48)	85(72-93)	100(3-100)	84/102	72	86(76-92)	18/102	9	50(26-74)	89(80-95)	43(22-66)
≥45 y	7/9	7	100	2/9	0	0	78	0	16/23	13	81(54-96)	7/23	2	29(4-71)	72(47-90)	40(5-85)

[1]Colposcopic impression and/or ZedScan result of CIN2+ of histologically confirmed CIN2+ cases.
[2]Colposcopic impression of CIN2+ of histologically confirmed CIN2+ cases.
[3]Colposcopic impression and ZedScan result less than CIN2 of histologically confirmed cases <CIN2.
[4]Colposcopic impression less than CIN2 of histologically confirmed cases <CIN2
AGC-FN: atypical glandular cells that favour neoplasia; AGC-NOS: atypical glandular cells not otherwise specified; ASC-H: atypical squamous cells that cannot exclude HSIL; ASC-US: atypical squamous cells of undetermined significance; CIN: cervical intraepithelial neoplasia; HSIL: high-grade squamous intraepithelial lesion; LSIL: low-grade squamous intraepithelial lesion; NPV: negative predictive value; PPV: positive predictive value; TZ: transformation zone
