# Supplementary material for: Added value of electrical impedance spectroscopy in adjunction of colposcopy: a prospective cohort study
Source: BMJ Open. 2023 Oct 29;13(10):e074921. doi: 10.1136/bmjopen-2023-074921 (PMC10619076; doi:10.1136/bmjopen-2023-074921)
Supplement: Supplementary data [file bmjopen-2023-074921supp004.pdf]

Table S3 Sensitivity and specificity of the electrical impedance spectroscopy cohort (EIS) and the reference cohort by cytology, TZ type and age group in identifying CIN2+, with corresponding risk differences and the risk ratios (RR) of sensitivity and specificity.

	EIS	Reference	Sensitivity			EIS	Reference	Specificity		
	Sensitivity	Sensitivity	Risk difference (95%)[1]	RR (95%)[1]	P value	Specificity	Specificity	Risk difference (95%)[1]	RR (95%)[1]	P value
ALL	94(90-97)	68(63-73)	0.26(0.20-0.31)	1.38(1.28-1.49)	<0.0001	34(29-39)	84(81-87)	-0.50(-0.56--0.45)	0.40(0.35-0.46)	<0.0001
ASC-US	94(70-100)	56(21-86)	0.38(0.04-0.73)	1.69(0.93-3.07)	0.0219	47(36-59)	97(91-99)	-0.49(-0.61--0.38)	0.49(0.39-0.62)	<0.0001
LSIL	77(61-89)	43(31-55)	0.34(0.16-0.51)	1.79(1.30-2.45)	0.0006	42(35-49)	92(89-95)	-0.51(-0.58--0.43)	0.45(0.38-0.53)	<0.0001
ASC-H	99(94-100)	63(54-71)	0.36(0.27-0.44)	1.57(1.38-1.78)	<0.0001	11(6-19)	65(54-74)	-0.53(-0.65--0.42)	0.17(0.10-0.30)	<0.0001
HSIL	100(95-100)	86(80-91)	0.14(0.08-0.19)	1.16(1.09-1.23)	0.0007	6(0-29)	46(31-61)	-0.40(-0.58--0.22)	0.13(0.02-0.89)	0.0033
AGC-NOS	67(9-99)	60(15-95)	0.07(-0.62-0.75)	1.11(0.38-3.25)	0.8504	44(24-65)	96(78-100)	-0.52(-0.73--0.30)	0.46(0.29-0.72)	0.0001
AGC-FN	50(1-99)	62(32-86)	-0.12(-0.86-0.63)	0.81(0.19-3.47)	0.7565	100(3-100)	25(1-81)	0.75(0.33-1.17)	4.0(0.73-21.84)	0.1709
TZ1	94(89-97)	67(61-73)	0.27(0.20-0.33)	1.40(1.27-1.53)	<0.0001	31(26-37)	84(80-88)	-0.53(-0.60--0.47)	0.37(0.31-0.44)	<0.0001
TZ2	95(87-99)	71(62-80)	0.24(0.14-0.34)	1.34(1.18-1.57)	0.0001	40(32-49)	84(79-88)	-0.44(-0.53--0.34)	0.48(0.38-0.59)	<0.0001
<30 y	93(84-98)	72(63-79)	0.22(0.12-0.32)	1.30(1.15-1.48)	0.0007	35(26-44)	77(70-83)	-0.42(-0.53--0.31)	0.45(0.35-0.59)	<0.0001
30-44 y	95(89-98)	68(62-75)	0.26(0.19-0.34)	1.39(1.25-1.53)	<0.0001	33(27-40)	86(81-90)	-0.53(-0.60--0.45)	0.39(0.32-0.47)	<0.0001
≥45 y	94(79-99)	59(43-73)	0.35(0.18-0.52)	1.59(1.23-2.07)	0.0008	35(24-47)	89(82-94)	-0.54(-0.66--0.42)	0.39(0.28-0.54)	<0.0001
HG cytology	99(96-100)	75(70-80)	0.24(0.19-0.29)	1.32(1.24-1.41)	<0.0001	11(6-18)	58(49-66)	-0.47(-0.56--0.37)	0.19(0.12-0.32)	<0.0001
LG cytology	81(69-90)	45(35-57)	0.36(0.21-0.50)	1.79(1.37-2.33)	<0.0001	43(38-49)	93(91-96)	-0.50(-0.56--0.44)	0.46(0.41-0.53)	<0.0001
1 biopsy	64(31-89)	36(13-65)	0.28(-0.10-0.66)	1.78(0.77-4.10)	0.1654	51(43-59)	99(94-100)	-0.48(-0.56--0.40)	0.51(0.44-0.60)	<0.0001
2 biopsies	90(81-96)	59(49-68)	0.31(0.19-0.42)	1.52(1.28-1.81)	<0.0001	23(17-30)	90(86-93)	-0.66(-0.73--0.59)	0.26(0.20-0.34)	<0.0001
≥3 biopsies	100	67(60-74)	0.33(0.26-0.40)	1.49(1.34-1.65)	<0.0001	0	67(59-75)	-0.67(-0.75--0.59)	0	<0.0001

[1]The values of risk difference >0 or the values of risk ratio >1 imply better/improved effect with ZedScan.

AGC-FN: atypical glandular cells that favour neoplasia; AGC-NOS: atypical glandular cells not otherwise specified; ASC-H: atypical squamous cells that cannot exclude HSIL; ASC-US: atypical squamous cells of undetermined significance; CIN: cervical intraepithelial neoplasia; HG: high grade; HSIL: high-grade squamous intraepithelial lesion; LG: low grade; LSIL: low-grade squamous intraepithelial lesion; TZ: transformation zone
