# Supplementary material for: Added value of electrical impedance spectroscopy in adjunction of colposcopy: a prospective cohort study
Source: BMJ Open. 2023 Oct 29;13(10):e074921. doi: 10.1136/bmjopen-2023-074921 (PMC10619076; doi:10.1136/bmjopen-2023-074921)
Supplement: Supplementary data [file bmjopen-2023-074921supp005.pdf]

Table S4 Average number of biopsies by cytology in the electrical impedance spectroscopy (EIS) cohort and in the reference cohort, including only the colposcopists who performed colposcopies in both cohorts.

	EIS cohort	Reference cohort
	Average number of biopsies	
ASC-US	1.7	2.3
LSIL	1.8	2.2
ASC-H	2.0	2.7
HSIL	2.3	2.8

ASC-US: atypical squamous cells of undetermined significance; ASC-H: atypical squamous cells that cannot exclude HSIL; HSIL: high-grade squamous intraepithelial lesion LSIL: low-grade squamous intraepithelial lesion
