# Supplementary material for: Added value of electrical impedance spectroscopy in adjunction of colposcopy: a prospective cohort study
Source: BMJ Open. 2023 Oct 29;13(10):e074921. doi: 10.1136/bmjopen-2023-074921 (PMC10619076; doi:10.1136/bmjopen-2023-074921)
Supplement: Author's manuscript [file bmjopen-2023-074921.draft_revisions.pdf]

BMJ Open is committed to open peer review. As part of this commitment we make the peer review history of every article we publish publicly available.

When an article is published we post the peer reviewers' comments and the authors' responses online. We also post the versions of the paper that were used during peer review. These are the versions that the peer review comments apply to.

The versions of the paper that follow are the versions that were submitted during the peer review process. They are not the versions of record or the final published versions. They should not be cited or distributed as the published version of this manuscript.

BMJ Open is an open access journal and the full, final, typeset and author-corrected version of record of the manuscript is available on our site with no access controls, subscription charges or pay-per-view fees (http://bmjopen.bmj.com).

If you have any questions on BMJ Open's open peer review process please email info.bmjopen@bmj.com

BMJ Open

Added value of electrical impedance spectroscopy in adjunction of colposcopy: a prospective cohort study

Journal:	*BMJ Open*
Manuscript ID	bmjopen-2023-074921
Article Type:	Original research
Date Submitted by the Author:	20-Apr-2023
Complete List of Authors:	Bergqvist, Laura; Helsinki University Central Hospital, Department of Obstetrics and Gynecology; Helsingin yliopisto<br>Heinonen, Annu; Helsinki University Central Hospital, Department of Obstetrics and Gynecology; Helsingin yliopisto<br>Carcopino, Xavier; IRD, Department of Obstetrics and Gynecology<br>Redman, Charles ; Royal Stoke University Hospital, Department of Obstetrics and Gynecology<br>Aro, Karoliina; Helsinki University Central Hospital, Department of Obstetrics and Gynecology; Helsingin yliopisto<br>Kiviharju, Mari; Helsinki University Central Hospital, Department of Obstetrics and Gynecology; Helsingin yliopisto<br>Virtanen, Seppo; Helsinki University Central Hospital, Department of Obstetrics and Gynecology; Helsingin yliopisto<br>Omar, Pirjo-Liisa; Helsinki University Central Hospital, Department of Obstetrics and Gynecology; Helsingin yliopisto<br>Kotaniemi-Talonen, Laura; Tampere University, Department of Obstetrics and Gynecology; Tampere University Hospital, Department of Obstetrics and Gynecology<br>Louvanto, Karolina; Tampere University, Department of Obstetrics and Gynecology; Tampere University Hospital, Department of Obstetrics and Gynecology<br>Nieminen, Pekka; Helsingin Yliopisto; Helsinki University Central Hospital, Department of Obstetrics and Gynecology<br>Kalliala, Ilkka ; Helsinki University Central Hospital Department of Obstetrics and Gynaecology; Imperial College London Department of Metabolism Digestion and Reproduction
Keywords:	Colposcopy < GYNAECOLOGY, Gynaecological oncology < GYNAECOLOGY, Community gynaecology < GYNAECOLOGY

SCHOLARONE™
Manuscripts

I, the Submitting Author has the right to grant and does grant on behalf of all authors of the Work (as defined in the below author licence), an exclusive licence and/or a non-exclusive licence for contributions from authors who are: i) UK Crown employees; ii) where BMJ has agreed a CC-BY licence shall apply, and/or iii) in accordance with the terms applicable for US Federal Government officers or employees acting as part of their official duties; on a worldwide, perpetual, irrevocable, royalty-free basis to BMJ Publishing Group Ltd ("BMJ") its licensees and where the relevant Journal is co-owned by BMJ to the co-owners of the Journal, to publish the Work in this journal and any other BMJ products and to exploit all rights, as set out in our licence.

The Submitting Author accepts and understands that any supply made under these terms is made by BMJ to the Submitting Author unless you are acting as an employee on behalf of your employer or a postgraduate student of an affiliated institution which is paying any applicable article publishing charge ("APC") for Open Access articles. Where the Submitting Author wishes to make the Work available on an Open Access basis (and intends to pay the relevant APC), the terms of reuse of such Open Access shall be governed by a Creative Commons licence – details of these licences and which Creative Commons licence will apply to this Work are set out in our licence referred to above.

Other than as permitted in any relevant BMJ Author's Self Archiving Policies, I confirm this Work has not been accepted for publication elsewhere, is not being considered for publication elsewhere and does not duplicate material already published. I confirm all authors consent to publication of this Work and authorise the granting of this licence.

For peer review only

Added value of electrical impedance spectroscopy in adjunction of colposcopy: a prospective cohort study

Laura Bergqvist,[1] Annu Heinonen,[1] Xavier Carcopino,[2] Charles Redman,[3] Karoliina Aro,[1] Mari Kiviharju,[1] Seppo Virtanen,[1] Pirjo-Liisa Omar,[1] Laura Kotaniemi-Talonen,[4,5] Karolina Louvanto,[4,5] Pekka Nieminen,*[1] Ilkka Kalliala*[1,6]

[1]Department of Obstetrics and Gynecology, University of Helsinki and Helsinki University Hospital, 00029 Helsinki, Finland

[2]Department of Obstetrics and Gynecology, Hôpital Nord, APHM, Aix-Marseille University (AMU), Univ Avignon, CNRS, IRD, IMBE UMR 7263, 13397, Marseille, France

[3]Formerly Consultant Gynecological Oncologist, University Hospital of North Midlands, Stoke-on-Trent

[4] Department of Obstetrics and Gynecology, Faculty of Medicine and Health Technology, Tampere University, 33100 Tampere, Finland

[5]Department of Obstetrics and Gynecology, Tampere University Hospital, 33100 Tampere, Finland

[6] Department of Metabolism, Digestion and Reproduction and Department of Surgery and Cancer, Faculty of Medicine, Imperial College London, London SW7 2AZ, UK

*Equal contribution

Correspondence: Laura Bergqvist, MD; Department of Obstetrics and Gynecology, University of Helsinki and Helsinki University Hospital, Haartmaninkatu 2, 00290 Helsinki, Finland; fax +358947174801; e-mail laura.bergqvist@hus.fi

For peer review only - http://bmjopen.bmj.com/site/about/guidelines.xhtml

Abstract (247)

Objective: To assess whether electrical impedance spectroscopy (EIS) as an adjunctive technology enhances the performance of colposcopy.

Design: Prospective cohort study.

Setting: University Hospital colposcopy clinic.

Participants: Colposcopy with EIS for 647 women and conventional colposcopy for 962 women.

Interventions: Comparison of the performance of colposcopy by referral cervical cytology in two cohorts, with and without EIS as an adjunctive technology.

Outcome measures: Prevalence of cervical intraepithelial neoplasia grade 2 or worse (CIN2+), diagnostic testing accuracy to detect CIN2+ with and without EIS and their relative differences between cohorts.

Results: The prevalence of CIN2+ varied between the cohorts according to referral cytology: 17.0% after abnormal squamous cells of unknown significance (ASC-US) referral cytology in EIS cohort and 9.1% in the reference cohort, 16.5% and 18.9% after low-grade squamous intraepithelial lesion (LSIL), 44.3% and 58.2% after atypical squamous cells, cannot exclude HSIL (ASC-H), and 81.9% and 77.0% after high-grade squamous intraepithelial lesion (HSIL) cytology, respectively. Sensitivity to detect CIN2+ was higher in the EIS cohort, varying from 1.79 (95% CI 1.30-2.45) after LSIL referral cytology to 1.16 (95% CI 1.09-1.23) after HSIL referral cytology, with correspondingly lower specificity after any referral cytology.

Conclusions: Colposcopy with EIS had overall higher sensitivity but lower specificity to detect CIN2+ than conventional colposcopy. CIN2+ prevalence rates were, however, not consistently higher in the EIS cohort, suggesting innate differences between the cohorts or truly lower detection rates of CIN2+ for EIS, highlighting the need for randomized controlled trials on the effectiveness of EIS.

Keywords: Electrical impedance spectroscopy (EIS), colposcopy, cervical intraepithelial neoplasia (CIN), cervical cytology, sensitivity, specificity

For peer review only - http://bmjopen.bmj.com/site/about/guidelines.xhtml

Strengths and limitations of this study

1. The performance and prevalence results of electrical impedance spectroscopy in adjunction with colposcopy were stratified by referral cytology.
2. The results were compared to a cohort with conventional colposcopy of the same colposcopy clinic representing similar populations.
3. The prevalence of CIN2+ was based on the first visit histological data.
4. The results offer information only in women with transformation zone type 1-2.

For peer review only - http://bmjopen.bmj.com/site/about/guidelines.xhtml

Introduction

During the next decades, the incidence and prevalence of high-grade cervical disease will decrease in the developed countries due to human papillomavirus (HPV) vaccination programs [1, 2] and transition to primary high-risk HPV (hrHPV)-DNA test -based screening.[3] Consequently, colposcopy will become more challenging due to resulting lower positive predictive value. Therefore, to detect those in need of treatment, it will be essential to correctly identify the high-grade lesions and take biopsies at representative locations. Also, reliable means to rule out high-grade lesions without excessive number of biopsies or frequently repeated tests or colposcopies are needed.

The sensitivity and specificity of colposcopy in identifying uterine cervical high-grade precancerous lesions have been previously reported to vary between 66% to 80% and 63% to 95%, respectively.[4-7] Furthermore, the probability of detecting a high-grade disease at colposcopy is affected by the referral cytology, being higher after high-grade squamous intraepithelial lesion (HSIL) cytology than after atypical squamous cells of undetermined significance (ASC-US) or low-grade squamous intraepithelial lesion (LSIL) cytology results.[8]

ZedScan (Zillico Ltd.)[9] is a hand-held device using electrical impedance spectroscopy (EIS) in identifying cervical pathology.[10] It is designed to provide guidance to colposcopist in biopsy taking by indicating the most abnormal cervical tissue area.[10] ZedScan measures the electrical properties of the cervical epithelium to differentiate pre-cancerous and cancerous tissue from normal epithelium.[10-12] The area with the most abnormal impedance is reported visually, aiding the colposcopist in targeting biopsies.

The sensitivity of colposcopy has been suggested to increase with the use of EIS [10, 13-16] even in women with low probability of high-grade cervical disease and with minor colposcopic changes, as its use is independent of visual findings in colposcopy.[12, 17] The developers of the technology have been involved in most of the published studies. In women with persistent hrHPV positivity without cervical cytological changes, EIS has detected additional cases of cervical intraepithelial neoplasia grade 2 or worse (CIN2+) compared to women without EIS examination.[17] The benefit of EIS seems to vary depending on the

For peer review only

For peer review only - http://bmjopen.bmj.com/site/about/guidelines.xhtml

referral cervical cytology, being most useful in terms of finding extra cases of CIN2+ in women with low-grade referral cervical cytology.[13, 14, 16] NICE guidelines recommend further research on EIS.[18]

Our objective was to assess, stratified according to referral cytology, whether EIS combined with colposcopy increases the diagnostic testing accuracy of CIN2+ compared to conventional colposcopy in women referred to colposcopy for abnormal cervical cytology.

Methods

Participants

All women (n=1609) in this study were examined between 2013-21 at the outpatient colposcopy clinic of Helsinki University Hospital for a new referral for abnormal cytology. We included women if their cervical transformation zone (TZ) was type 1 or 2 (TZ1-2) and the information on both colposcopic impression and histopathological results were available. Exclusion criteria were transformation zone type 3 (TZ3) and pregnancy. Women referred for persistent hrHPV positivity without cytological changes were excluded due to the lack of sufficient control cohort as high-risk HPV testing as a part of primary screening was implemented in Helsinki region only in 2019.

The EIS cohort consisted of 647 women with colposcopy and ZedScan examination successfully performed between September 2018 and August 2021. The cohort was collected prospectively with non-consecutive patient recruitment. Under the study period ZedScan equipment was available at the colposcopy and used at the decision of the individual colposcopist. EIS examinations were done according to the manufacturer's protocol and all colposcopists had an adequate training prior using the device. If active bleeding during colposcopy occurred, the EIS procedure was omitted.

We could not directly compare the performance of colposcopy alone against colposcopy with ZedScan as an adjunctive tool using only the EIS cohort, as these two events were not truly independent of each other in the routine clinical setting applied here. Therefore, we used a previously collected prospective cohort of 962 patients examined with conventional colposcopy in the colposcopy clinic of Helsinki University

For peer review only - http://bmjopen.bmj.com/site/about/guidelines.xhtml

For peer review only

Hospital, Finland, between 2013 and July 2017 as the reference cohort (ISRCTN10933736),[19] with all women fulfilling the inclusion criteria included. Only the primary colposcopy after referral and its histological results were included in both cohorts.

Abnormal cervical cytology results were categorized according to the Bethesda system as ASC-US or worse. Histological results were reported according to WHO 2003, 2013 and 2020 classification. The evaluation of histopathological specimens, biopsies, and large loop excision of the transformation zone (LLETZ) cones, was done by the gynaecological histopathologists of Helsinki University Hospital. The most severe histological diagnosis of all biopsies or LLETZ was recorded.

Clinical procedures

All participants had a colposcopic examination with the application of acetic acid to the cervix. Subsequently, participants in the EIS cohort underwent a ZedScan examination. ZedScan readings were made from 10 to 12 points clockwise around the cervix. On the Zedscan reading, red colour points out the area with the highest probability of high-grade disease, amber colour indicates possible high-grade areas and the absence of high-grade disease is indicated with green colour. In the EIS cohort, cervical biopsy sites were determined by the colposcopist based on both ZedScan results and colposcopic impression. The most severe histological diagnosis of all biopsies was recorded.

Random biopsies were not routinely taken in either of the cohorts. Colposcopy examination in both cohorts was based on Finnish Current Care Guidelines.[20] Five percent acetic acid and Lugol's iodine were available at the discretion of individual colposcopist to assess the abnormal cervical areas for biopsy. The colposcopic impression was recorded as high-grade, low-grade, or normal. Immediate LLETZ at initial visit ('select and treat'-approach) was performed when evaluated necessary according to Finnish Current Care Guidelines: HSIL referral cytology with a colposcopic impression of CIN2+ entitled to perform LLETZ at the initial colposcopy with consent from the patient.[20] After cervical cytology with glandular atypia favouring neoplasia (AGC-FN) the Finnish Current Care Guidelines recommends immediate LLETZ irrespective of the age of the referred woman.[20]

For peer review only - http://bmjopen.bmj.com/site/about/guidelines.xhtml

Data analysis

We compared the prevalence of histologically confirmed CIN2+ lesions between the EIS and reference cohorts and calculated the sensitivity, specificity, positive and negative predictive values for colposcopy in both cohorts for the detection of CIN2+ lesions, both overall and stratified according to the referral cervical cytology. The positive test result for EIS cohort was defined as suspected presence of CIN2+ either by ZedScan and/or via colposcopic inspection. The test result was negative if both the colposcopic impression and ZedScan agreed on low-grade lesion or normal cervical finding, i.e. absence of CIN2+ lesion. In the reference cohort, positive test result was defined as a colposcopic impression of CIN2+ while negative test result was defined as the absence of changes suggesting CIN2+ lesions. The most advanced histopathological result of the biopsies or LLETZ specimen taken at the initial visit were used as a reference standard in both cohorts. Women without biopsies and with negative ZedScan result and normal colposcopic impression as well as low-grade referral were considered true negatives. Even though colposcopy and EIS examination were not truly independent tests in the setting used, we still performed a sensitivity analysis within the EIS cohort and assessed separately diagnostic testing accuracy of colposcopy and EIS in that cohort alone as well.

Risk ratio and risk difference were used to compare the sensitivity and specificity between the EIS and reference cohorts. The *p*-values <0.05 were considered statistically significant. All statistical analyses were performed using STATA/SE 15 (StataCorp, College Station TX, USA) and all statistical tests used were two-sided.

Results

There were 1027 eligible women with adequate colposcopy and Zedscan examination performed In the EIS cohort. Altogether 68 women with other referral reasons than abnormal cervical cytology, 215 women with follow-up colposcopy visits and 97 women with missing data were excluded. In total, 647 women with new colposcopy referrals of abnormal cytology were included in the analysis (Table 1). Of all ZedScan procedures 75% were conducted by three individual colposcopists. The reference cohort included 1383

For peer review only - http://bmjopen.bmj.com/site/about/guidelines.xhtml

Table 1. Characteristics of the electrical impedance spectroscopy (EIS) cohort and the reference cohort.

	EIS cohort		Reference cohort	
	n=647	%	n=962	%
Mean age	35.7		35.4	
Std.deviation, Range	9.3(20.3-76.4)		9.6(19.2-67.8)	
**Referral cervical cytology**				
ASC-US	94	14.5	99	10.3
LSIL	236	36.5	381	39.6
ASC-H	192	29.7	237	24.6
HSIL	94	14.5	200	20.8
AGC-NOS	28	4.3	28	2.9
AGC-FN	3	0.5	17	1.8
	647	100	962	100
**Age**				
<30 y	175	27.0	295	30.7
30-44 y	366	56.6	495	51.5
>45 y	106	16.4	172	17.9
	647	100	962	100
**TZ type**				
TZ type 1	446	68.9	620	64.4
TZ type 2	201	31.1	342	35.6
	647	100	962	100
**Biopsies and LLETZ**				
No biopsy	22	3.4	10	1.0
1 biopsy	165	25.5	109	11.3
2 biopsies	263	40.6	420	43.7
3 biopsies	83	12.8	257	26.7
4 biopsies	1	0.2	43	4.5
5 biopsies	0	0	5	0.5
LLETZ	113	17.5	118	12.3
	647	100	962	100
**Histology**				
No biopsy	22	3.4	10	1.0
Normal histology	222	34.3	248	25.8
CIN1 (LSIL)	181	28.0	313	32.5
CIN2 (HSIL)	95	14.7	210	21.8
CIN3 (HSIL)	107	16.5	154	16.0
Glandular atypia	1	0.2	5	0.7
AIS	14	2.2	15	1.6
Adenocarcinoma	3	0.5	3	0.3
Sq. cell carcinoma	2	0.3	4	0.4
	647	100	962	100

Std. deviation: standard deviation; EIS: electrical impedance spectroscopy; CIN: cervical intraepithelial neoplasia; TZ: transformation zone; ASC-US: atypical squamous cells of undetermined significance; LSIL: low-grade squamous intraepithelial lesion; HSIL: high-grade squamous intraepithelial lesion; ASC-H: atypical squamous cells that cannot exclude HSIL; AGC-NOS: atypical glandular cells not otherwise specified; AGC-FN: atypical glandular cells that favor neoplasia; AIS: adenocarcinoma in situ; sq. cell carcinoma: squamous cell carcinoma; LLETZ: large loop excision of the transformation zone

For peer review only

For peer review only - http://bmjopen.bmj.com/site/about/guidelines.xhtml

eligible women. Of these, 86 women were excluded due to other referral reasons than abnormal cervical cytology, 174 for having TZ3, 143 for missing relevant clinical data and 18 for pregnancy. As a result, a total of 962 women fulfilled the inclusion criteria (Table 1).

At least one biopsy was taken or imminent LLETZ made in 625 (96.6%) women in the EIS cohort and 952 (99.0%) in the reference cohort. Only one biopsy was taken from one quarter of women 165 (25.5%) in the EIS cohort and among 109 (11.3%) in the reference cohort, whereas twenty-two (3.4%) women in the EIS cohort and 10 (1.0%) in the reference cohort had no biopsy. The average number of biopsies was 1.8 if at least one biopsy was taken in the EIS cohort and 2.3 in the reference cohort. (Table 1)

Altogether 222 (34.3%) women in the EIS cohort had CIN2+, including 5 (0.8%) cervical carcinomas and 14 (2.2%) adenocarcinoma in situ cases. In the reference cohort 391 (40.6%) women had CIN2+, including 7 (0.7%) cervical carcinomas and 15 (1.6%) adenocarcinoma in situ cases. (Table 1). The prevalence of CIN2+ was higher in the reference cohort among those referred for LSIL or ASC-H cytology, whereas the prevalence of CIN2+ was higher in the EIS cohort after ASC-US and HSIL referral cytology (Table 2).

In the EIS cohort the overall sensitivity to detect CIN2+ was 94% (95% CI 90-97%) with corresponding specificity of 34% (95% CI 29-39%) (Table 2). The sensitivity varied according to referral cytology, being the lowest, 77%, for LSIL cytology (95% CI 61-89%) and the highest for HSIL cytology with 100% sensitivity (95% CI 95-100%) (Table 2). The specificity was lowest for HSIL cytology, 6% (95% CI 0-29%), and highest for ASC-US, 47% (95% CI 36-59%). EIS missed 3 low-grade referral cases of CIN2+ identified by the colposcopist (two cases if CIN2 and one CIN3). Colposcopic impression was less than CIN2 in 43 CIN2+ cases that were detected by ZedScan. A total of 13 cases (5.9%) of CIN2+ were missed by both ZedScan and the colposcopist (biopsies still taken due to suspicion of low-grade lesion), including two adenocarcinoma in situ cases and eleven high-grade lesions (nine CIN2 and two CIN3 cases).

In the reference cohort, the overall sensitivity to detect CIN2+ was 68% (95% CI 63-73%) with corresponding specificity of 84% (95% CI 81-87%) (Table 2). The sensitivity to detect CIN2+ by colposcopic impression of CIN2+ was the lowest after LSIL cytology, 43%, and the highest after HSIL cytology, 86%

For peer review only

For peer review only - http://bmjopen.bmj.com/site/about/guidelines.xhtml

Table 2. Sensitivity, specificity, negative and positive predictive value of the electrical impedance spectroscopy (EIS) cohort and the reference cohort for the detection of CIN2+ lesions by cervical cytology, TZ type and age group.

	EIS (n=647)									Reference cohort (n=962)								
	CIN2+/n	CIN2%	Colpo+ZS CIN2+[1]	Sensitivity	<CIN2/n	Colpo +ZS <CIN2[3]	Specificity	PPV	NPV	CIN2+/n	CIN2%	Colpo CIN2+[2]	Sensitivity	<CIN2/n	Colpo <CIN2[4]	Specificity	PPV	NPV
ASC-US	16/94	17.0	15	94(70-100)	78/94	37	47(36-59)	27(16-40)	97(86-100)	9/99	9.1	5	56(21-86)	90/99	87	97(91-99)	63(25-92)	96(89-99)
LSIL	39/236	16.5	30	77(61-89)	197/236	82	42(35-49)	21(14-28)	90(82-95)	72/381	18.9	31	43(31-55)	309/381	285	92(89-95)	56(42-70)	87(83-91)
ASC-H	85/192	44.3	84	99(94-100)	107/192	12	11(6-19)	47(39-55)	92(64-100)	138/237	58.2	87	63(54-71)	99/237	64	65(54-74)	71(62-79)	56(46-65)
HSIL	77/94	81.9	77	100(95-100)	17/94	1	6(0-29)	83(74-90)	100(3-100)	154/200	77.0	133	86(80-91)	46/200	21	46(31-61)	84(78-90)	50(34-66)
AGC-NOS	3/28	10.7	2	67(9-99)	25/28	11	44(24-65)	13(2-38)	92(62-100)	5/28	17.9	3	60(15-95)	23/28	22	96(78-100)	75(19-99)	92(73-99)
AGC-FN	2/3	66.7	1	50(1-99)	1/3	1	100(3-100)	100(3-100)	50(1-99)	13/17	76.5	8	62(32-86)	4/17	1	25(1-81)	73(39-94)	17(0-64)
ALL	222/647	34.3	209	94(90-97)	425/647	144	34(29-39)	43(38-47)	92(86-96)	391/962	40.6	267	68(63-73)	571/962	480	84(81-87)	75(70-79)	80(76-83)
TZ1	156/446	35.0	146	94(89-97)	290/446	90	31(26-37)	42(37-48)	90(82-95)	279/620	45.0	187	67(61-73)	341/620	287	84(80-88)	78(72-83)	76(71-80)
TZ2	66/201	32.8	63	96(87-99)	135/201	54	40(32-49)	44(36-52)	95(85-99)	112/342	32.7	80	71(62-80)	230/342	193	84(79-88)	68(59-77)	86(81-90)
<30 y	60/175	34.3	56	93(84-98)	115/175	40	35(26-44)	43(34-52)	91(78-98)	134/295	45.4	96	72(63-79)	161/295	124	77(70-83)	72(64-80)	77(69-83)
30-44 y	131/366	35.8	124	95(89-98)	235/366	78	33(27-40)	44(38-50)	92(84-97)	211/495	42.6	144	68(62-75)	284/495	244	86(81-90)	78(72-84)	79(74-83)
>45 y	31/106	29.2	29	94(79-99)	75/106	26	35(24-47)	37(27-49)	93(77-99)	46/172	26.7	27	59(43-73)	126/172	112	89(82-94)	66(49-80)	86(78-91)
HG cytology	164/289	56.7	162	99(96-100)	125/289	14	11(6-18)	59(53-65)	88(62-98)	305/454	67.2	228	75(70-80)	149/454	86	58(49-66)	78(73-83)	53(45-61)
LG cytology	58/358	16.2	47	81(69-90)	300/358	130	43(38-49)	22(16-28)	92(87-96)	86/508	16.9	39	45(35-57)	422/508	394	93(91-96)	58(46-70)	89(86-92)
ASC-H, HSIL	162/286	56.6	161	99(97-100)	124/286	13	11(6-17)	59(53-65)	93(66-100)	292/437	66.8	220	75(70-80)	145/437	85	59(50-67)	79(73-83)	54(46-62)
ASC-US,LSIL	55/330	16.7	45	82(69-91)	275/330	119	43(37-49)	22(17-29)	92(86-96)	81/480	16.9	36	44(33-56)	399/480	372	93(90-96)	57(44-70)	89(86-92)
Glandular	5/31	16.1	3	60(15-95)	26/31	12	46(27-67)	18(4-43)	86(57-98)	18/45	40.0	11	61(36-83)	27/45	23	85(66-96)	73(45-92)	77(58-90)
1 biopsy	11/165	6.7	7	64(31-89)	154/165	78	51(43-59)	8(4-17)	95(88-99)	14/109	12.8	5	36(13-65)	95/109	94	99(94-100)	83(36-100)	91(84-96)
2 biopsies	78/263	29.7	70	90(81-96)	185/263	43	23(17-30)	33(27-40)	84(71-93)	112/420	26.7	66	59(49-68)	308/420	276	90(86-93)	67(57-77)	86(81-89)
≥3 biopsies	43/84	51.2	43	100	41/84	0	0	51	0	168/305	55.1	113	67(60-74)	137/305	92	67(59-75)	72(64-78)	63(54-70)
LLETZ	90/113	79.6	89	99(94-100)	23/113	1	4(0-22)	80(72-87)	50(1-99)	97/118	82.2	83	86(77-92)	21/118	8	38(18-62)	87(78-93)	36(17-59)

EIS: electrical impedance spectroscopy; CIN: cervical intraepithelial neoplasia; TZ: transformation zone; PPV: positive predictive value; NPV: negative predictive value; ASC-US: atypical squamous cells of undetermined significance; LSIL: low-grade squamous intraepithelial lesion; HSIL: high-grade squamous intraepithelial lesion; ASC-H: atypical squamous cells that cannot exclude HSIL; AGC-NOS: atypical glandular cells not otherwise specified; AGC-FN: atypical glandular cells that favor neoplasia; LLETZ: large loop excision of the transformation zone; HG: high grade; LG: low grade

[1]Colposcopic impression and/or ZedScan result of CIN2+ of histologically confirmed CIN2+ cases.

[2]Colposcopic impression of CIN2+ of histologically confirmed CIN2+ cases.

[3]Colposcopic impression and ZedScan result less than CIN2 of histologically confirmed cases <CIN2.

[4]Colposcopic impression less than CIN2 of histologically confirmed cases <CIN2

For peer review only

For peer review only - http://bmjopen.bmj.com/site/about/guidelines.xhtml

(Figure 1, Table 2). Overall, the colposcopic impression was less than CIN2+ in 31.7% (124/391) of CIN2+ cases and biopsies were taken due to suspicion of a low-grade lesion. Results stratified according to TZ type, age, and referral cytology are presented in Table S1.

Compared to the referral cohort, the sensitivity to detect CIN2+ was higher in the EIS cohort overall, with risk ratio (RR) of 1.38 (95% CI 1.28-1.49), and after LSIL, ASC-H and HSIL referral cervical cytologies (Table 3). TZ 1 and taking two or more biopsies were associated with higher observed sensitivity (Table 3). Specificity was correspondingly lower in the EIS cohort overall as well as when stratified according to referral cytology (Table 3).

In the EIS cohort, colposcopic impression of high-grade disease (CIN2+) was present with EIS indicating the presence of CIN2+ in 73.4% of all histologically confirmed CIN2+ cases. In the sensitivity analysis within the EIS cohort, colposcopy alone was indicative for the presence of CIN2+ in 166 of 222 CIN2+ cases (74.8%) and ZedScan in 206 of 222 (92.8%) of CIN2+ cases, suggesting an additional 40 cases (24.1%) detected by ZedScan only. The additional cases increased the detection of CIN2+ from 30 to 44 in women with low-grade cytology and from 136 to 162 in women with high-grade cytology (Figure 1). The sensitivity to detect CIN2+ by colposcopy alone according to referral cytology was otherwise similar between the cohorts, except for women with ASC-H cervical cytology the colposcopy alone in the EIS cohort seemed to detect more CIN2+ cases (p=0.02) (Figure 1).

For peer review only - http://bmjopen.bmj.com/site/about/guidelines.xhtml

Table 3. Sensitivity and specificity of the electrical impedance spectroscopy cohort (EIS) and the reference cohort by cytology, TZ type and age group in identifying CIN2+, with corresponding risk differences and the risk ratios of sensitivity and specificity.

	EIS	Reference	Sensitivity			EIS	Reference	Specificity		
	Sensitivity	Sensitivity	Risk difference (95%)[1]	RR (95%)[1]	p.	Specificity	Specificity	Risk difference (95%)[1]	RR (95%)[1]	p.
ASC-US	94(70-100)	56(21-86)	0.38(0.04-0.73)	1.69(0.93-3.07)	0.0219	47(36-59)	97(91-99)	-0.49(-0.61--0.38)	0.49(0.39-0.62)	<0.0001
LSIL	77(61-89)	43(31-55)	0.34(0.16-0.51)	1.79(1.30-2.45)	0.0006	42(35-49)	92(89-95)	-0.51(-0.58--0.43)	0.45(0.38-0.53)	<0.0001
ASC-H	99(94-100)	63(54-71)	0.36(0.27-0.44)	1.57(1.38-1.78)	<0.0001	11(6-19)	65(54-74)	-0.53(-0.65--0.42)	0.17(0.10-0.30)	<0.0001
HSIL	100(95-100)	86(80-91)	0.14(0.08-0.19)	1.16(1.09-1.23)	0.0007	6(0-29)	46(31-61)	-0.40(-0.58--0.22)	0.13(0.02-0.89)	0.0033
AGC-NOS	67(9-99)	60(15-95)	0.07(-0.62-0.75)	1.11(0.38-3.25)	0.8504	44(24-65)	96(78-100)	-0.52(-0.73--0.30)	0.46(0.29-0.72)	0.0001
AGC-FN	50(1-99)	62(32-86)	-0.12(-0.86-0.63)	0.81(0.19-3.47)	0.7565	100(3-100)	25(1-81)	0.75(0.33-1.17)	4.0(0.73-21.84)	0.1709
ALL	94(90-97)	68(63-73)	0.26(0.20-0.31)	1.38(1.28-1.49)	<0.0001	34(29-39)	84(81-87)	-0.50(-0.56--0.45)	0.40(0.35-0.46)	<0.0001
TZ1	94(89-97)	67(61-73)	0.27(0.20-0.33)	1.40(1.27-1.53)	<0.0001	31(26-37)	84(80-88)	-0.53(-0.60--0.47)	0.37(0.31-0.44)	<0.0001
TZ2	96(87-99)	71(62-80)	0.24(0.14-0.34)	1.34(1.18-1.57)	0.0001	40(32-49)	84(79-88)	-0.44(-0.53--0.34)	0.48(0.38-0.59)	<0.0001
					0.0007					
<30 y	93(84-98)	72(63-79)	0.22(0.12-0.32)	1.30(1.15-1.48)		35(26-44)	77(70-83)	-0.42(-0.53--0.31)	0.45(0.35-0.59)	<0.0001
30-44 y	95(89-98)	68(62-75)	0.26(0.19-0.34)	1.39(1.25-1.53)	<0.0001	33(27-40)	86(81-90)	-0.53(-0.60--0.45)	0.39(0.32-0.47)	<0.0001
≥45 y	94(79-99)	59(43-73)	0.35(0.18-0.52)	1.59(1.23-2.07)	0.0008	35(24-47)	89(82-94)	-0.54(-0.66--0.42)	0.39(0.28-0.54)	<0.0001
HG cytology	99(96-100)	75(70-80)	0.24(0.19-0.29)	1.32(1.24-1.41)	<0.0001	11(6-18)	58(49-66)	-0.47(-0.56--0.37)	0.19(0.12-0.32)	<0.0001
LG cytology	81(69-90)	45(35-57)	0.36(0.21-0.50)	1.79(1.37-2.33)	<0.0001	43(38-49)	93(91-96)	-0.50(-0.56--0.44)	0.46(0.41-0.53)	<0.0001
1 biopsy	64(31-89)	36(13-65)	0.28(-0.10-0.66)	1.78(0.77-4.10)	0.1654	51(43-59)	99(94-100)	-0.48(-0.56--0.40)	0.51(0.44-0.60)	<0.0001
2 biopsies	90(81-96)	59(49-68)	0.31(0.19-0.42)	1.52(1.28-1.81)	<0.0001	23(17-30)	90(86-93)	-0.66(-0.73--0.59)	0.26(0.20-0.34)	<0.0001
≥3 biopsies	100	67(60-74)	0.33(0.26-0.40)	1.49(1.34-1.65)	<0.0001	0	67(59-75)	-0.67(-0.75--0.59)	0	<0.0001

EIS: electrical impedance spectroscopy; CIN: cervical intraepithelial neoplasia; TZ: transformation zone; ASC-US: atypical squamous cells of undetermined significance; LSIL: low-grade squamous intraepithelial lesion; HSIL: high-grade squamous intraepithelial lesion; ASC-H: atypical squamous cells that cannot exclude HSIL; AGC-NOS: atypical glandular cells not otherwise specified; AGC-FN: atypical glandular cells that favor neoplasia; HG: high grade; LG: low grade

[1]The values of risk difference >0 or the values of risk ratio >1 imply better/improved effect with ZedScan.

For peer review only

For peer review only - http://bmjopen.bmj.com/site/about/guidelines.xhtml

239 **Discussion**

240 We compared the performance of colposcopy in detecting CIN2+ according to referral cervical cytology
241 with and without EIS as an adjunctive technology. Colposcopy combined with EIS seemed to have a higher
242 sensitivity, but a lower specificity compared to conventional colposcopy, regardless of the referral cervical
243 cytology. The prevalence of CIN2+ lesions was higher in the EIS cohort after ASC-US and HSIL referral, but
244 lower after LSIL and ASC-H cervical cytology. The average number of biopsies was lower in the EIS cohort.

245

246 Overall, EIS performed well with a high sensitivity (94%) but had a low specificity (34%) consistent with the
247 previous studies.[13, 14, 16] Here, the sensitivity might have been overestimated in both cohorts as the
248 true positive result was based on histology data at first visit only and lesions missed at first visit and
249 detected during the follow-up were not included in either cohort. Still, this would not affect the estimates
250 of relative performance. The sensitivity (68%) and specificity (84%) of colposcopy in the reference cohort
251 was as well in line with existing data.[5, 7, 21]

252 The increased detection of CIN2+ cases by EIS has been reported as most pronounced in women with low-
253 grade cytology [13, 14, 16] or with high-risk HPV positivity without cytological changes.[16, 17] In our study,
254 additional cases of CIN2+ detected by EIS were also most frequent among low-grade referrals.
255 Furthermore, the sensitivity to detect CIN2+ with EIS was higher in most cervical cytology groups (ASC-US,
256 LSIL, ASC-H, HSIL) compared to colposcopy alone. Only within HSIL cytology EIS combined with colposcopy
257 detected all CIN2+ cases. In women with other referral cytology (ASC-US, LSIL, ASC-H) there were cases of
258 CIN2+ that EIS did not detect, but where biopsy of CIN2+ was warranted based on colposcopic diagnosis.
259 Nevertheless, missed cases of CIN2+ were even more frequent in the reference cohort, where more CIN2+
260 lesions were detected in biopsies with colposcopic impression of CIN1 or lower. Contrary to expectations,
261 the prevalence of CIN2+ was higher in EIS cohort only after ASC-US and HSIL referral cytology. One
262 explanation for lower prevalence of CIN2+ lesions in the EIS cohort after LSIL and ASC-H cytology could be
263 that routine practice in Finland is to take biopsies also from low-grade lesions, rather than to abstain from

For peer review only

For peer review only - http://bmjopen.bmj.com/site/about/guidelines.xhtml

taking biopsies when CIN2+ lesions are not colposcopically suspected. Biopsies even from mild acetowhite lesions are important in excluding a high-grade disease as the sensitivity of colposcopy to detect CIN2+ is far from 100%. Such biopsies could well have been more frequent without than with EIS as an additional confirmation on suspected absence of CIN2+. This is supported by the observation that two or more biopsies were taken from 54% of women in the EIS cohort, whereas up to 75% of women in the reference cohort had at least two biopsies. Multiple biopsies are known to increase the sensitivity of colposcopy as at least small lesions can easily be missed.[22, 23] In women with low-grade referral cervical cytology, a single biopsy has shown to be insufficient to rule out a high-grade disease.[24] A British survey has also reported experienced colposcopists to take mostly two biopsies in diagnosing high-grade disease.[25] The average number of biopsies in the EIS cohort was higher (1.84) compared to previous reports (1.07 and 1.51),[23, 14] but still lower than in the reference cohort (2.3).

Our observation of overall fewer biopsies along with fewer CIN2+ lesions detected in the EIS cohort can either indicate a true difference in CIN2+ prevalence between the cohorts, selection bias towards using EIS preferably on patients in whom CIN2+ lesion is not clearly present, or that CIN2+ lesions could have been missed in the EIS cohort, especially after LSIL and ASC-H referral cytology. If lesions were missed, it could possibly be due to a higher biopsy threshold in the EIS cohort, as indicated by lower number of biopsies. Without longitudinal data we still cannot be certain whether prevalent CIN2+ cases were indeed more frequently missed at the first visit in the EIS cohort. The prevalence of CIN2+ in EIS cohort in women with high-grade cytology (ASC-H and HSIL) is below previous observations (56.6% vs. 79.1-84.0%).[13, 16] However, when restricted to only women with HSIL referral cervical cytology or low-grade (ASC-US and LSIL) cytology, the prevalence for CIN2+ here did not differ from previous reports.[13, 16] Cytological diagnoses may well vary between cytopathologists as well as between countries and this possible difference in classification might also explain the observed difference in CIN2+ prevalence, especially after ASC-H cytology.[26] The longitudinal data on EIS results are scarce. In women referred with low-grade cytology, the future risk of CIN2+ was increased in up to 36 months follow-up if both colposcopic impression and EIS results were indicative for CIN2+ compared with women with other combinations of

For peer review only

these two parameters, suggesting that EIS might provide new information on the future risk of high-grade disease.[27]

Strengths and limitations

Most previous studies have compared the performance of EIS as an adjunctive technology for colposcopy against conventional colposcopy within the cohort where EIS was used, even though in clinical setting EIS is not a truly independent measurement from colposcopy. To our knowledge this is the first report on the performance of EIS as an adjunctive technology for colposcopy stratified according to referral cytology and compared to an external reference cohort. Even though our cohorts were collected at different time periods, they both represent women in the same catchment area referred to colposcopy due to abnormal cervical cytology. All colposcopies were performed in the same clinic by experienced colposcopists. Furthermore, none of the authors of this work have financial conflicts of interest with the technology studied. Our study also has some limitations. When the cervical transformation zone is not fully visible, TZ type 3, ZedScan technology cannot be reliably applied and the results are not applicable to this population. CIN2+ lesions could well have been missed in both cohorts since the results are based on data collected on the initial visit. However, complete certainty of the histology would have required LLETZ for all participants which would not have been ethically just.

Conclusions

Colposcopy with EIS has a higher sensitivity and a lower specificity in identifying CIN2+ compared to conventional colposcopy, irrespective of cervical cytology. EIS can, therefore, be assumed to be of clinical benefit in colposcopy, particularly in women with low-grade cervical cytology where the prevalence of CIN2+ is low. We also observed an overall lower prevalence of CIN2+ lesions in the EIS cohort compared to a reference cohort with conventional colposcopy. The performance of EIS as an adjunctive technology for colposcopy has not been previously compared by cytology to an external reference cohort. While the observation of lower CIN2+ rate could be explained by different CIN2+ prevalence between the cohorts or selection bias, the finding is important and warrants further research, especially along with the observed

For peer review only

For peer review only - http://bmjopen.bmj.com/site/about/guidelines.xhtml

lower number of biopsies in the EIS cohort. Adjunctive technologies are likely to become increasingly appealing in colposcopy, as the prevalence of high-grade cervical lesions is declining. Randomised controlled trials comparing EIS with a conventional colposcopy, including women referred due to persistent HPV infection without cytological changes are warranted. Before such further evidence, firm recommendations on applicability of EIS as an adjunctive technology for colposcopy cannot be made.

Figure 1. Numbers and rates of CIN2+ lesions detected in the electrical impedance spectroscopy cohort (EIS) and in the reference cohort according to referral cytology. (A) Numbers and rates of CIN2+ detected by ZedScan alone and reference cohort stratified according to referral cytology (B) Numbers and rates of CIN2+ detected by colposcopy alone in EIS and reference cohorts stratified according to referral cytology. Numbers of patients are given in the columns.

Contributors

PN and IK were responsible for the conceptualisation and design of the study as well as methodology. XC, KL, LKT contributed to conceptualisation. LB performed the statistical analysis with the aid of IK. LB, PN, MK, PLO, SV and AH were responsible for data collection. LB drafted the original manuscript and IK, PN, CR, XC, KL, LKT, AH and KA participated in writing, reviewing and editing. All authors listed qualify for authorship and approved the final version of the paper.

Funding

This work was supported by the Academy of Finland (IK), the Finnish Medical Foundation (IK), and Finnish State Research Funding (IK).

Competing interests None declared.

Patient and public involvement Patients and/or the public were not involved in the design, or conduct, or reporting, or dissemination plans of this research.

Patient consent for publication Not required.

For peer review only

For peer review only - http://bmjopen.bmj.com/site/about/guidelines.xhtml

Ethics approval This study and data collection on patients where EIS was used was considered as a service evaluation and therefore a separate ethical approval was not required as per consultation with Helsinki-Uusimaa Hospital District Ethical Committee. For the historical reference cohort an ethical approval was received from Helsinki-Uusimaa Hospital District Ethical Committee (ref. no. 130/13/03/03/2013).

Data availability statement Data are available upon reasonable request.

For peer review only

For peer review only - http://bmjopen.bmj.com/site/about/guidelines.xhtml

References:

1 Munro A, Gillespie C, Cotton S, et al. The impact of human papillomavirus type on colposcopy performance in women offered HPV immunisation in a catch-up vaccine programme: a two-centre observational study. *BJOG* 2017;124(9):1394–401.

2 Lehtinen M, Pimenoff VN, Nedjai B, et al. Assessing the risk of cervical neoplasia in the post-HPV vaccination era. *Int J Cancer* 2022;(June):1–9.

3 Rebolj M, Rimmer J, Denton K, et al. Primary cervical screening with high risk human papillomavirus testing: Observational study. *BMJ* 2019;364:l240.

4 Zuchna C, Hager M, Tringler B, et al. Diagnostic accuracy of guided cervical biopsies: A prospective multicenter study comparing the histopathology of simultaneous biopsy and cone specimen. *Am J Obstet Gynecol* 2010;203(4):321.e1-321.e6. Available: http://dx.doi.org/10.1016/j.ajog.2010.05.033

5 Cantor SB, Cárdenas-Turanzas M, Cox DD, et al. Accuracy of colposcopy in the diagnostic setting compared with the screening setting. *Obstet Gynecol* 2008;111(1):7–14.

6 Underwood M, Arbyn M, Parry-Smith W, et al. Accuracy of colposcopy-directed punch biopsies: a systematic review and meta-analysis. *BJOG* 2012;119(11):1293–1301.

7 Brown BH, Tidy JA. The diagnostic accuracy of colposcopy – A review of research methodology and impact on the outcomes of quality assurance. *Eur J of Obstet Gynecol Repro Biol* 2019;240(2019):182–6. Available: https://doi.org/10.1016/j.ejogrb.2019.07.003

8 Demarco M, Lorey TS, Fetterman B, et al. Risks of CIN 2 +, CIN 3 +, and Cancer by Cytology and Human Papillomavirus Status: The Foundation of Risk-Based Cervical Screening Guidelines. *J Low Genit Tract Dis* 2017;21(4):261–7.

9 Zilico Ltd. Manchester: Available: https://zilico.co.uk

10 Tidy JA, Brown BH, Healey TJ, et al. Accuracy of detection of high-grade cervical intraepithelial neoplasia using electrical impedance spectroscopy with colposcopy. *BJOG* 2013;120(4):400–11.

For peer review only

For peer review only - http://bmjopen.bmj.com/site/about/guidelines.xhtml

11 Brown BH, Tidy JA, Boston K, et al. Relation between tissue structure and imposed electrical current flow in cervical neoplasia. *The Lancet* 2000 Mar 11;355(9207):892–5. Available: https://doi.org/10.1016/S0140-6736(99)09095-9

12 Balasubramani L, Brown BH, Healey J, et al. The detection of cervical intraepithelial neoplasia by electrical impedance spectroscopy: The effects of acetic acid and tissue homogeneity. *Gynecol Oncol* 2009;115(2):267–71. Available: http://dx.doi.org/10.1016/j.ygyno.2009.08.010

13 Tidy JA, Brown BH, Lyon RE, et al. Are colposcopy and electrical impedance spectroscopy complementary when used to detect high-grade cervical neoplasia? *Eur J Gynaecol Oncol* 2018;39(1):70–5.

14 Muszynski C, Dupont E, Vaysse B, et al. The impact of using electrical impedance spectroscopy (ZedScan) on the performance of colposcopy in diagnosing high grade squamous lesions of the cervix. *J Gynecol Obstet Hum Reprod* 2017;46(9):669–73. Available: http://dx.doi.org/10.1016/j.jogoh.2017.08.007

15 Homola W, Fuchs T, Baranski P, et al. Use of electrical impedance spectroscopy as an adjunct to colposcopy in a pathway of cervical intraepithelial neoplasia diagnostics. *Ginekol Pol* 2019;90(11):628–32.

16 Tidy JA, Brown BH. Increased detection of high grade CIN, when using electrical impedance spectroscopy as an adjunct to routine colposcopy, is maintained when used across international boundaries: Prospective data from nine European countries. *Eur J Obstet and Gynecol Repro Biol* 2022;275(May):41–5. Available: https://doi.org/10.1016/j.ejogrb.2022.05.025

17 Tidy JA, Lyon R, Ellis K, et al. The impact of age and high-risk human papillomavirus (hrHPV) status on the prevalence of high-grade cervical intraepithelial neoplasia (CIN2+) in women with persistent hrHPV-positive, cytology-negative screening samples: a prospective cohort study. *BJOG* 2020;127(10):1260–7.

18 National Institute for Health and Care Excellence. NICE Adjunctive colposcopy technologies for assessing suspected cervical abnormalities: the DYSIS colposcope with DYSISmap and the Zedscan I. 2018. Available: https://www.nice.org.uk/guidance/dg32 [Accessed 22 Jan 2022].

For peer review only

For peer review only - http://bmjopen.bmj.com/site/about/guidelines.xhtml

19 Aro K, Nieminen P, Louvanto K, et al. Gynecologic Oncology Age-specific HPV type distribution in high-grade cervical disease in screened and unvaccinated women. *Gynecol Oncol* 2019;154(2):354–9. Available: https://doi.org/10.1016/j.ygyno.2019.05.024

20 Working group set up by the Finnish Medical Society Duodecim and the Finnish Colposcopy Association. Cytological Changes in the Cervix, Vagina and Vulva. Current Care Guidelines. The Finnish Medical Society Duodecim, 2021. Available: www.kaypahoito.fi [Accessed 17 Jan 2022].

21 van der Marel J, van Baars R, Quint WGV, et al. The impact of human papillomavirus genotype on colposcopic appearance: A cross-sectional analysis. *BJOG* 2014;121(9):1117–26.

22 Pretorius RG, Belinson JL, Burchette RJ, et al. Regardless of skill, performing more biopsies increases the sensitivity of colposcopy. *J Low Genit Tract Dis* 2011;15(3):180–8.

23 Wentzensen N, Walker JL, Gold MA, et al. Multiple biopsies and detection of cervical cancer precursors at colposcopy. *J Clin Oncol* 2015;33(1):83–9.

24 Moss EL, Hadden P, Douce G, et al. Is the colposcopically directed punch biopsy a reliable diagnostic test in women with minor cytological lesions? *J Low Genit Tract Dis* 2012;16(4):421–6.

25 Myriokefalitaki E, Redman CWE, Potdar N, et al. The Use of the Colposcopically Directed Punch Biopsy in Clinical Practice: A Survey of British Society of Colposcopy and Cervical Pathology (BSCCP)-Accredited Colposcopists. *J Low Genit Tract Dis* 2016;20(3):234–8.

26 Sherman ME, Castle PE, Solomon D. Cervical cytology of atypical squamous cells-cannot exclude high-grade squamous intraepithelial lesion (ASC-H): Characteristics and histologic outcomes. *Cancer* 2006 Oct 25;108(5):298–305.

27 Brown BH, Highfield PE, Tidy JA. Prognostic value of electrical impedance spectroscopy (EIS) when used as an adjunct to colposcopy - A longitudinal study. *J Electr Bioimp* 2020;11(1):81–6.

For peer review only - http://bmjopen.bmj.com/site/about/guidelines.xhtml

(A) (B)

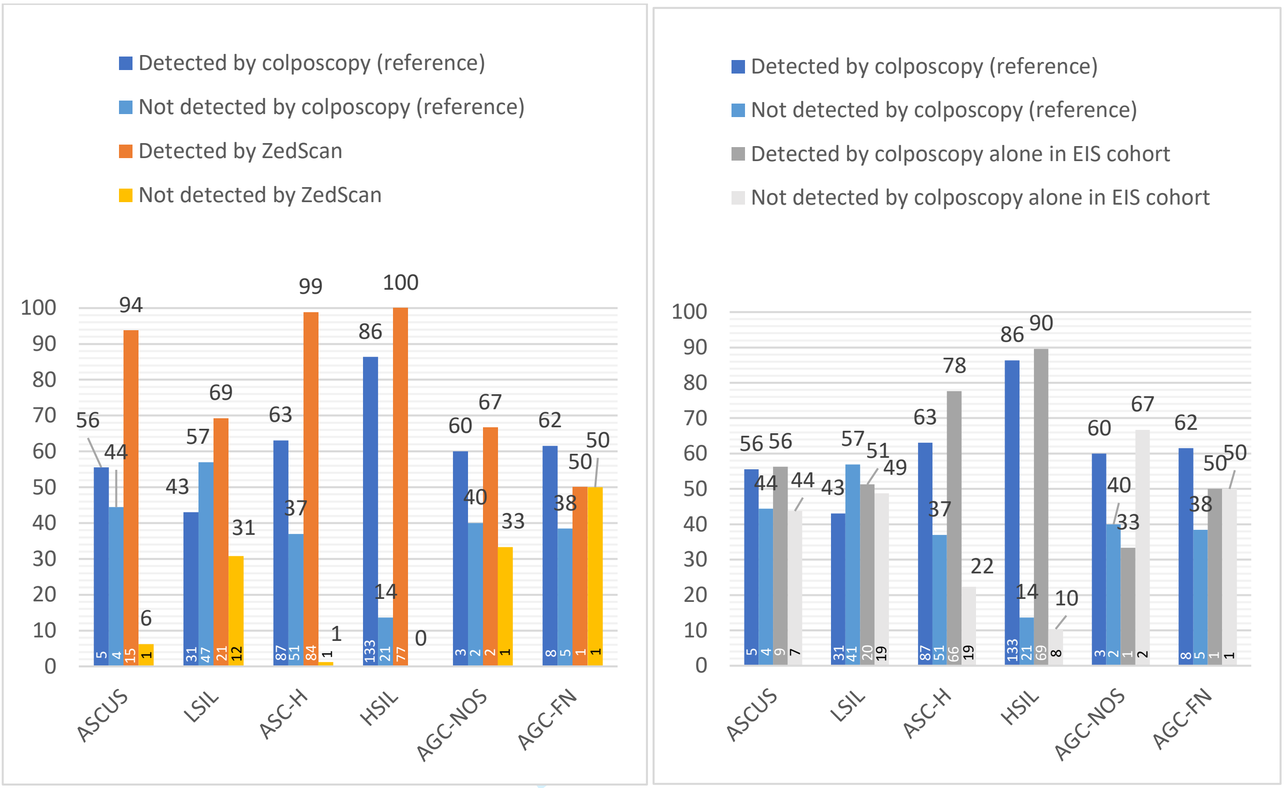

review only

Table S1. Sensitivity, specificity, positive and negative predictive value of the electrical impedance spectroscopy (EIS) cohort and the reference cohort for the detection of CIN2+ lesions within different cervical cytology by TZ type and age group.

	EIS cohort (n=647)								Reference cohort (n=962)							
		Colpo+ZS[1]			Colpo+ZS[3]					Colpo[2]			Colpo[4]			
	CIN2+/n	CIN2+	Sensitivity	<CIN/n	<CIN2	Specificity	PPV	NPV	CIN2+/n	CIN2+	Sensitivity	<CIN2/n	<CIN2	Specificity	PPV	NPV
ASC-US	16/94	15	94(70-100)	78/94	37	47(36-59)	27(16-40)	97(86-100)	9/99	5	56(21-86)	90/99	87	97(91-99)	63(25-92)	96(89-99)
TZ1	11/66	10	91(59-100)	55/66	24	44(30-58)	24(12-40)	96(80-100)	7/57	3	43(10-82)	50/57	47	94(84-99)	50(12-88)	92(81-98)
TZ2	5/28	5	100(48-100)	23/28	13	57(35-77)	33(12-62)	100(75-100)	2/42	2	100(16-100)	40/42	40	100(91-100)	100(16-100)	100(91-100)
<30 y	6/28	5	83(36-100)	22/28	12	55(32-76)	33(12-62)	92(64-100)	1/43	0	0(0-98)	42/43	39	93(81-99)	0(0-71)	98(87-100)
30-44 y	10/52	10	100(69-100)	42/52	21	50(34-66)	32(17-51)	100(84-100)	7/28	4	57(18-90)	21/28	21	100(84-100)	100(40-100)	88(68-97)
>45 y	0/14	0	0	14/14	4	29	0	100	1/28	1	100(3-100)	27/28	27	100(87-100)	100(3-100)	100(87-100)
LSIL	39/236	30	77(61-89)	197/236	82	42(35-49)	21(14-28)	90(82-95)	72/381	31	43(31-55)	309/381	285	92(89-95)	56(42-70)	87(83-91)
TZ1	26/157	20	77(56-91)	131/157	53	41(32-49)	20(13-30)	90(79-96)	53/235	23	43(30-58)	182/235	169	93(88-96)	64(46-79)	85(79-90)
TZ2	13/79	10	77(46-95)	66/79	29	44(32-57)	21(11-36)	91(75-98)	19/146	8	42(20-67)	127/146	116	91(85-96)	42(20-67)	91(85-96)
<30 y	4/39	1	25(1-81)	35/39	21	60(42-76)	7(0-32)	88(68-97)	17/79	9	53(28-77)	62/79	54	87(76-94)	53(28-77)	87(76-94)
30-44 y	28/153	24	86(67-96)	125/153	46	37(28-46)	23(16-33)	92(81-98)	46/224	19	41(27-57)	178/224	166	93(89-97)	61(42-78)	86(80-91)
>45 y	7/44	5	71(29-96)	37/44	15	41(25-58)	19(6-38)	88(64-99)	9/78	3	33(8-70)	69/78	65	94(86-98)	43(10-82)	92(83-97)
ASC-H	85/192	84	99(94-100)	107/192	12	11(6-19)	47(39-55)	92(64-100)	138/237	87	63(54-71)	99/237	64	65(54-74)	71(62-79)	56(46-65)
TZ1	57/134	56	98(91-100)	77/134	6	8(3-16)	44(35-53)	86(42-100)	100/167	64	64(54-73)	67/167	42	63(50-74)	72(61-81)	54(42-65)
TZ2	28/58	28	100(88-100)	30/58	6	20(8-39)	54(40-68)	100(54-100)	38/70	23	61(43-76)	32/70	22	69(50-84)	70(51-84)	60(42-75)
<30 y	24/72	24	100(86-100)	48/72	5	10(4-23)	36(25-49)	100(48-100)	57/90	34	60(46-72)	33/90	18	55(36-72)	69(55-82)	44(29-60)
30-44 y	46/90	45	98(89-100)	44/90	4	9(3-22)	53(42-64)	80(28-100)	67/120	45	67(55-78)	53/120	37	70(56-82)	74(61-84)	63(49-75)
>45 y	15/30	15	100(78-100)	15/30	3	20(4-48)	56(35-75)	100(29-100)	14/27	8	57(29-82)	13/27	9	69(39-91)	67(35-90)	60(32-84)
HSIL	77/94	77	100(95-100)	17/94	1	6(0-29)	83(74-90)	100(3-100)	154/200	133	86(80-91)	46/200	21	46(31-61)	84(78-90)	50(34-66)
TZ1	58/67	58	100	9/67	0	0	87	0	104/131	88	85(76-91)	27/131	15	56(35-75)	88(80-94)	48(30-67)
TZ2	19/27	19	100(82-100)	8/27	1	13(0-53)	73(52-88)	100(3-100)	50/69	45	90(78-97)	19/69	6	32(13-57)	78(65-88)	55(23-83)
<30 y	25/31	25	100	6/31	0	0	81	0	54/75	48	89(77-96)	21/75	10	48(26-70)	81(69-90)	63(35-85)
30-44 y	45/54	45	100(92-100)	9/54	1	11(0-48)	85(72-93)	100(3-100)	84/102	72	86(76-92)	18/102	9	50(26-74)	89(80-95)	43(22-66)
>45 y	7/9	7	100	2/9	0	0	78	0	16/23	13	81(54-96)	7/23	2	29(4-71)	72(47-90)	40(5-85)

EIS: electrical impedance spectroscopy; CIN: cervical intraepithelial neoplasia; TZ: transformation zone; PPV: positive predictive value; NPV: negative predictive value; ASC-US: atypical squamous cells of undetermined significance; LSIL: low-grade squamous intraepithelial lesion; HSIL: high-grade squamous intraepithelial lesion; ASC-H: atypical squamous cells that cannot exclude HSIL; AGC-NOS: atypical glandular cells not otherwise specified; AGC-FN: atypical glandular cells that favor neoplasia; LLETZ: large loop excision of the transformation zone; HG: high grade; LG: low grade

[1]Colposcopic impression and/or ZedScan result of CIN2+ of histologically confirmed CIN2+ cases.

[2]Colposcopic impression of CIN2+ of histologically confirmed CIN2+ cases.

[3]Colposcopic impression and ZedScan result less than CIN2 of histologically confirmed cases <CIN2.

[4]Colposcopic impression less than CIN2 of histologically confirmed cases <CIN2

For peer review only

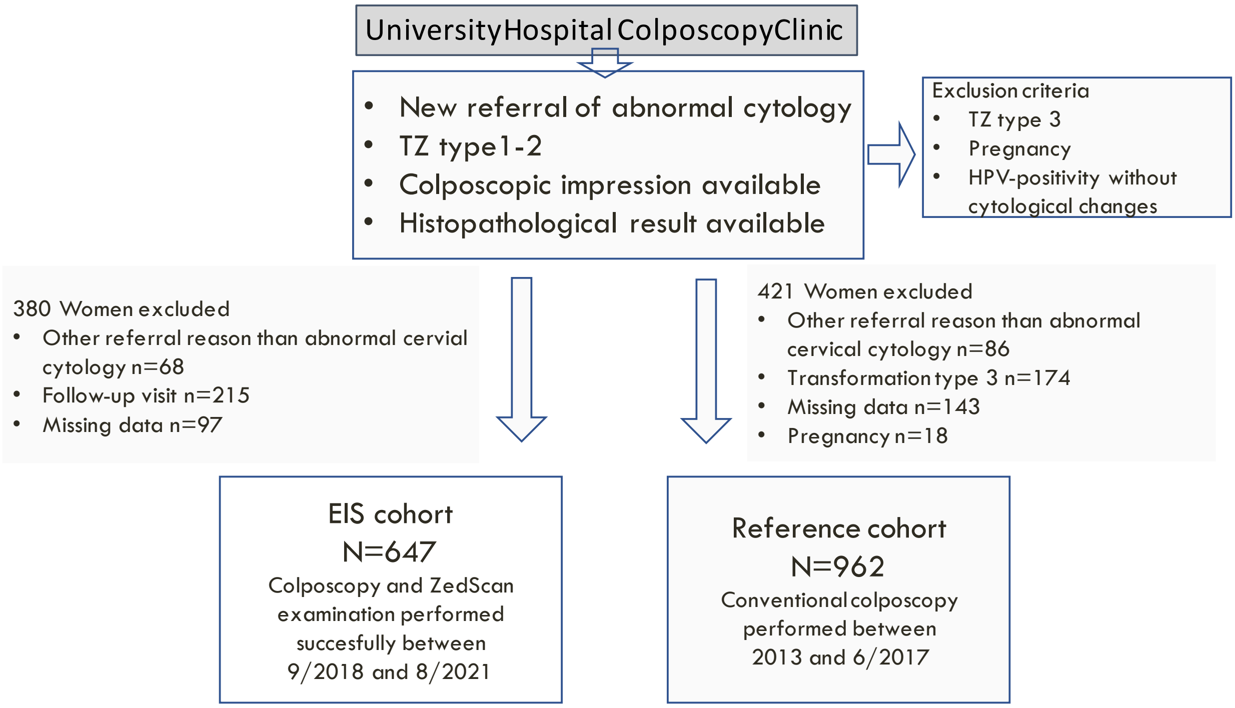

Figure S1. Flow-chart of the study comparing the performance of colposcopy by referral cervical cytology in two cohorts with and without electrical impedance spectroscopy as an adjunctive technology.

Section & Topic	No	Item	Reported on page #
**TITLE OR ABSTRACT**			
	**1**	Identification as a study of diagnostic accuracy using at least one measure of accuracy (such as sensitivity, specificity, predictive values, or AUC)	2
**ABSTRACT**			
	**2**	Structured summary of study design, methods, results, and conclusions (for specific guidance, see STARD for Abstracts)	2
**INTRODUCTION**			
	**3**	Scientific and clinical background, including the intended use and clinical role of the index test	4
	**4**	Study objectives and hypotheses	4
**METHODS**			
Study design	**5**	Whether data collection was planned before the index test and reference standard were performed (prospective study) or after (retrospective study)	5
Participants	**6**	Eligibility criteria	5-6
	**7**	On what basis potentially eligible participants were identified (such as symptoms, results from previous tests, inclusion in registry)	5
	**8**	Where and when potentially eligible participants were identified (setting, location and dates)	5-6
	**9**	Whether participants formed a consecutive, random or convenience series	5-6
Test methods	**10a**	Index test, in sufficient detail to allow replication	6
	**10b**	Reference standard, in sufficient detail to allow replication	6
	**11**	Rationale for choosing the reference standard (if alternatives exist)	5-6
	**12a**	Definition of and rationale for test positivity cut-offs or result categories of the index test, distinguishing pre-specified from exploratory	7
	**12b**	Definition of and rationale for test positivity cut-offs or result categories of the reference standard, distinguishing pre-specified from exploratory	7
	**13a**	Whether clinical information and reference standard results were available to the performers/readers of the index test	6
	**13b**	Whether clinical information and index test results were available to the assessors of the reference standard	5-6
Analysis	**14**	Methods for estimating or comparing measures of diagnostic accuracy	7
	**15**	How indeterminate index test or reference standard results were handled	5-6
	**16**	How missing data on the index test and reference standard were handled	5-6
	**17**	Any analyses of variability in diagnostic accuracy, distinguishing pre-specified from exploratory	7
	**18**	Intended sample size and how it was determined	5-6
**RESULTS**			
Participants	**19**	Flow of participants, using a diagram	Figure S1, submitted as a separate file
	**20**	Baseline demographic and clinical characteristics of participants	8, Table 1
	**21a**	Distribution of severity of disease in those with the target condition	8, Table 1
	**21b**	Distribution of alternative diagnoses in those without the target condition	8, Table 1
	**22**	Time interval and any clinical interventions between index test and reference standard	5-6
Test results	**23**	Cross tabulation of the index test results (or their distribution) by the results of the reference standard	8,10,12 Table1-3
	**24**	Estimates of diagnostic accuracy and their precision (such as 95% confidence intervals)	10,12 Table 2-3
	**25**	Any adverse events from performing the index test or the reference standard	NA
**DISCUSSION**			
	**26**	Study limitations, including sources of potential bias, statistical uncertainty, and generalisability	13-15
	**27**	Implications for practice, including the intended use and clinical role of the index test	15-16
**OTHER INFORMATION**			
	**28**	Registration number and name of registry	6 ISRCTN for the reference cohort

For peer review only - http://bmjopen.bmj.com/site/about/guidelines.xhtml

For peer review only

	**29**	Where the full study protocol can be accessed	Submitted as a separate file
	**30**	Sources of funding and other support; role of funders	16

For peer review only

STARD 2015

AIM

STARD stands for "Standards for Reporting Diagnostic accuracy studies". This list of items was developed to contribute to the completeness and transparency of reporting of diagnostic accuracy studies. Authors can use the list to write informative study reports. Editors and peer-reviewers can use it to evaluate whether the information has been included in manuscripts submitted for publication.

EXPLANATION

A **diagnostic accuracy study** evaluates the ability of one or more medical tests to correctly classify study participants as having a **target condition.** This can be a disease, a disease stage, response or benefit from therapy, or an event or condition in the future. A medical test can be an imaging procedure, a laboratory test, elements from history and physical examination, a combination of these, or any other method for collecting information about the current health status of a patient.

The test whose accuracy is evaluated is called **index test.** A study can evaluate the accuracy of one or more index tests. Evaluating the ability of a medical test to correctly classify patients is typically done by comparing the distribution of the index test results with those of the **reference standard**. The reference standard is the best available method for establishing the presence or absence of the target condition. An accuracy study can rely on one or more reference standards.

If test results are categorized as either positive or negative, the cross tabulation of the index test results against those of the reference standard can be used to estimate the **sensitivity** of the index test (the proportion of participants *with* the target condition who have a positive index test), and its **specificity** (the proportion *without* the target condition who have a negative index test). From this cross tabulation (sometimes referred to as the contingency or "2x2" table), several other accuracy statistics can be estimated, such as the positive and negative **predictive values** of the test. Confidence intervals around estimates of accuracy can then be calculated to quantify the statistical **precision** of the measurements.

If the index test results can take more than two values, categorization of test results as positive or negative requires a **test positivity cut-off**. When multiple such cut-offs can be defined, authors can report a receiver operating characteristic (ROC) curve which graphically represents the combination of sensitivity and specificity for each possible test positivity cut-off. The **area under the ROC curve** informs in a single numerical value about the overall diagnostic accuracy of the index test.

The **intended use** of a medical test can be diagnosis, screening, staging, monitoring, surveillance, prediction or prognosis. The **clinical role** of a test explains its position relative to existing tests in the clinical pathway. A replacement test, for example, replaces an existing test. A triage test is used before an existing test; an add-on test is used after an existing test.

Besides diagnostic accuracy, several other outcomes and statistics may be relevant in the evaluation of medical tests. Medical tests can also be used to classify patients for purposes other than diagnosis, such as staging or prognosis. The STARD list was not explicitly developed for these other outcomes, statistics, and study types, although most STARD items would still apply.

DEVELOPMENT

This STARD list was released in 2015. The 30 items were identified by an international expert group of methodologists, researchers, and editors. The guiding principle in the development of STARD was to select items that, when reported, would help readers to judge the potential for bias in the study, to appraise the applicability of the study findings and the validity of conclusions and recommendations. The list represents an update of the first version, which was published in 2003.

More information can be found on http://www.equator-network.org/reporting-guidelines/stard.

For peer review only

Added value of electrical impedance spectroscopy in adjunction of colposcopy: a prospective cohort study

Journal:	*BMJ Open*
Manuscript ID	bmjopen-2023-074921.R1
Article Type:	Original research
Date Submitted by the Author:	17-Aug-2023
Complete List of Authors:	Bergqvist, Laura; Helsinki University Central Hospital, Department of Obstetrics and Gynecology; University of Helsinki<br>Heinonen, Annu; Helsinki University Central Hospital, Department of Obstetrics and Gynecology; University of Helsinki<br>Carcopino, Xavier; IRD, Department of Obstetrics and Gynecology<br>Redman, Charles ; Royal Stoke University Hospital, Department of Obstetrics and Gynecology<br>Aro, Karoliina; Helsinki University Central Hospital, Department of Obstetrics and Gynecology; University of Helsinki<br>Kiviharju, Mari; Helsinki University Central Hospital, Department of Obstetrics and Gynecology; University of Helsinki<br>Virtanen, Seppo; Helsinki University Central Hospital, Department of Obstetrics and Gynecology; University of Helsinki<br>Omar, Pirjo-Liisa; Helsinki University Central Hospital, Department of Obstetrics and Gynecology; University of Helsinki<br>Kotaniemi-Talonen, Laura; Tampere University Hospital, Department of Obstetrics and Gynecology; Tampere University, Faculty of Medicine and Health Technology<br>Louvanto, Karolina; Tampere University, Department of Obstetrics and Gynecology; Tampere University Hospital, Department of Obstetrics and Gynecology<br>Nieminen, Pekka; University of Helsinki; Helsinki University Central Hospital, Department of Obstetrics and Gynecology<br>Kalliala, Ilkka ; Helsinki University Central Hospital Department of Obstetrics and Gynaecology; Imperial College London Department of Metabolism Digestion and Reproduction
**Primary Subject Heading**:	Obstetrics and gynaecology
Secondary Subject Heading:	Obstetrics and gynaecology, Oncology
Keywords:	Colposcopy < GYNAECOLOGY, Gynaecological oncology < GYNAECOLOGY, Community gynaecology < GYNAECOLOGY

For peer review only - http://bmjopen.bmj.com/site/about/guidelines.xhtml

I, the Submitting Author has the right to grant and does grant on behalf of all authors of the Work (as defined in the below author licence), an exclusive licence and/or a non-exclusive licence for contributions from authors who are: i) UK Crown employees; ii) where BMJ has agreed a CC-BY licence shall apply, and/or iii) in accordance with the terms applicable for US Federal Government officers or employees acting as part of their official duties; on a worldwide, perpetual, irrevocable, royalty-free basis to BMJ Publishing Group Ltd ("BMJ") its licensees and where the relevant Journal is co-owned by BMJ to the co-owners of the Journal, to publish the Work in this journal and any other BMJ products and to exploit all rights, as set out in our licence.

The Submitting Author accepts and understands that any supply made under these terms is made by BMJ to the Submitting Author unless you are acting as an employee on behalf of your employer or a postgraduate student of an affiliated institution which is paying any applicable article publishing charge ("APC") for Open Access articles. Where the Submitting Author wishes to make the Work available on an Open Access basis (and intends to pay the relevant APC), the terms of reuse of such Open Access shall be governed by a Creative Commons licence – details of these licences and which Creative Commons licence will apply to this Work are set out in our licence referred to above.

Other than as permitted in any relevant BMJ Author's Self Archiving Policies, I confirm this Work has not been accepted for publication elsewhere, is not being considered for publication elsewhere and does not duplicate material already published. I confirm all authors consent to publication of this Work and authorise the granting of this licence.

For peer review only

Added value of electrical impedance spectroscopy in adjunction of colposcopy: a prospective cohort study

Laura Bergqvist,[1] Annu Heinonen,[1] Xavier Carcopino,[2] Charles Redman,[3] Karoliina Aro,[1] Mari Kiviharju,[1] Seppo Virtanen,[1] Pirjo-Liisa Omar,[1] Laura Kotaniemi-Talonen,[4,5] Karolina Louvanto,[4,5] Pekka Nieminen,*[1] Ilkka Kalliala*[1,6]

[1]Department of Obstetrics and Gynecology, University of Helsinki and Helsinki University Hospital, 00029 Helsinki, Finland

[2]Department of Obstetrics and Gynecology, Hôpital Nord, APHM, Aix-Marseille University (AMU), Univ Avignon, CNRS, IRD, IMBE UMR 7263, 13397, Marseille, France

[3]Formerly Consultant Gynecological Oncologist, University Hospital of North Midlands, Stoke-on-Trent

[4] Department of Obstetrics and Gynecology, Faculty of Medicine and Health Technology, Tampere University, 33100 Tampere, Finland

[5]Department of Obstetrics and Gynecology, Tampere University Hospital, 33100 Tampere, Finland

[6] Department of Metabolism, Digestion and Reproduction and Department of Surgery and Cancer, Faculty of Medicine, Imperial College London, London SW7 2AZ, UK

*Equal contribution

Correspondence: Laura Bergqvist, MD; Department of Obstetrics and Gynecology, University of Helsinki and Helsinki University Hospital, Haartmaninkatu 2, 00290 Helsinki, Finland; fax +358947174801; e-mail laura.bergqvist@hus.fi

For peer review only - http://bmjopen.bmj.com/site/about/guidelines.xhtml

Abstract

Objective: To assess whether electrical impedance spectroscopy (EIS) as an adjunctive technology enhances the performance of colposcopy.

Design: Prospective cohort study.

Setting: University Hospital colposcopy clinic.

Participants: Colposcopy with EIS for 647 women and conventional colposcopy for 962 women.

Interventions: Comparison of the performance of colposcopy by referral cervical cytology in two cohorts, with and without EIS as an adjunctive technology.

Outcome measures: Prevalence of cervical intraepithelial neoplasia grade 2 or worse (CIN2+), diagnostic testing accuracy to detect CIN2+ with and without EIS and their relative differences between cohorts.

Results: The prevalence of CIN2+ varied between the cohorts according to referral cytology: 17.0% after abnormal squamous cells of unknown significance (ASC-US) referral cytology in EIS cohort and 9.1% in the reference cohort, 16.5% and 18.9% after low-grade squamous intraepithelial lesion (LSIL), 44.3% and 58.2% after atypical squamous cells, cannot exclude HSIL (ASC-H), and 81.9% and 77.0% after high-grade squamous intraepithelial lesion (HSIL) cytology, respectively. Sensitivity to detect CIN2+ was higher in the EIS cohort, varying from 1.79 (95% CI 1.30-2.45) after LSIL referral cytology to 1.16 (95% CI 1.09-1.23) after HSIL referral cytology, with correspondingly lower specificity after any referral cytology.

Conclusions: Colposcopy with EIS had overall higher sensitivity but lower specificity to detect CIN2+ than conventional colposcopy. CIN2+ prevalence rates were, however, not consistently higher in the EIS cohort, suggesting innate differences between the cohorts or truly lower detection rates of CIN2+ for EIS, highlighting the need for randomized controlled trials on the effectiveness of EIS.

Keywords: Electrical impedance spectroscopy (EIS), colposcopy, cervical intraepithelial neoplasia (CIN), cervical cytology, sensitivity, specificity

For peer review only - http://bmjopen.bmj.com/site/about/guidelines.xhtml

Strengths and limitations of this study

1. The intervention and reference cohorts were both collected within the daily patient flow at the same colposcopy clinic.
2. The reference cohort was collected between 2013-2017 (n=962) and the EIS cohort between 2018-2021 (n=647).
3. The prevalence of CIN2+ in both cohorts was based on the histopathological data obtained at the first visit.
4. Diagnostic testing accuracy was calculated for the detection of CIN2+ in both cohorts.
5. We estimated the added value of electrical impedance spectroscopy compared to conventional colposcopy within and between cohorts stratified according to the referral cytology.

For peer review only

Introduction

During the next decades, the incidence and prevalence of high-grade cervical disease will decrease in the developed countries due to human papillomavirus (HPV) vaccination programs [1, 2] and transition to primary high-risk HPV (hrHPV)-DNA test -based screening.[3] Consequently, colposcopy will become more challenging due to resulting lower positive predictive value. Therefore, to detect those in need of treatment, it will be essential to correctly identify the high-grade lesions and take biopsies at representative locations. Also, reliable means to rule out high-grade lesions without excessive number of biopsies or frequently repeated tests or colposcopies are needed.

The sensitivity and specificity of colposcopy in identifying uterine cervical high-grade precancerous lesions have been previously reported to vary between 66% to 80% and 63% to 95%, respectively.[4-7] Furthermore, the probability of detecting a high-grade disease at colposcopy is affected by the referral cytology, being higher after high-grade squamous intraepithelial lesion (HSIL) cytology than after atypical squamous cells of undetermined significance (ASC-US) or low-grade squamous intraepithelial lesion (LSIL) cytology results.[8]

ZedScan (Zillico Ltd.)[9] is a hand-held device using electrical impedance spectroscopy (EIS) in identifying cervical pathology.[10] It is designed to provide guidance to colposcopist in biopsy taking by indicating the most abnormal cervical tissue area.[10] ZedScan measures the electrical properties of the cervical epithelium to differentiate pre-cancerous and cancerous tissue from normal epithelium.[10-12] The area with the most abnormal impedance is reported visually, aiding the colposcopist in targeting biopsies.

The sensitivity of colposcopy has been suggested to increase with the use of EIS [10, 13-17] even in women with low probability of high-grade cervical disease and with minor colposcopic changes, as its use is independent of visual findings in colposcopy.[12, 18, 19] The developers of the technology have been involved in most of the published studies. In women with persistent hrHPV positivity without cervical cytological changes, EIS has detected additional cases of cervical intraepithelial neoplasia grade 2 or worse (CIN2+) compared to women without EIS examination.[18] The benefit of EIS seems to vary depending on

For peer review only

the referral cervical cytology, being most useful in terms of finding extra cases of CIN2+ in women with low-grade referral cervical cytology.[13, 14, 16, 17] NICE guidelines recommend further research on EIS.[20]

Our objective was to assess, stratified according to referral cytology, whether EIS combined with colposcopy increases the diagnostic testing accuracy of CIN2+ compared to conventional colposcopy in women referred to colposcopy for abnormal cervical cytology.

Methods

Participants

All women (n=1609) in this study were examined between 2013-21 at the outpatient colposcopy clinic of Helsinki University Hospital for a new referral for abnormal cytology. We included women if their cervical transformation zone (TZ) was type 1 or 2 (TZ1-2) and the information on both colposcopic impression and histopathological results were available. Exclusion criteria were transformation zone type 3 (TZ3), previous history of cervical cancer or large loop excision of the transformation zone (LLETZ) and pregnancy. Women referred for persistent hrHPV positivity without cytological changes were excluded due to the lack of sufficient control cohort as high-risk HPV testing as a part of primary screening was implemented in Helsinki region only in 2019.

The EIS cohort consisted of 647 women with colposcopy and ZedScan examination successfully performed between September 2018 and August 2021. The cohort was collected prospectively with non-consecutive patient recruitment. Under the study period ZedScan equipment was available at the colposcopy and used at the decision of the individual colposcopist. EIS examinations were done according to the manufacturer's protocol and all colposcopists had an adequate training prior using the device. If active bleeding during colposcopy occurred, the EIS procedure was omitted.

We could not directly compare the performance of colposcopy alone against colposcopy with ZedScan as an adjunctive tool using only the EIS cohort, as these two events were not truly independent of each other in the routine clinical setting applied here. Therefore, we used a previously collected prospective cohort of

For peer review only

962 patients examined with conventional colposcopy in the colposcopy clinic of Helsinki University Hospital, Finland, between 2013 and July 2017 as the reference cohort (ISRCTN10933736),[21] with all women fulfilling the inclusion criteria included. Only the primary colposcopy after referral and its histological results were included in both cohorts.

Abnormal cervical cytology results were categorized according to the Bethesda system as ASC-US or worse. Histological results were reported according to WHO 2003, 2013 and 2020 classification. The evaluation of histopathological specimens, biopsies, and LLETZ cones, was done by the gynaecological histopathologists of Helsinki University Hospital. The most severe histological diagnosis of all biopsies or LLETZ was recorded.

Clinical procedures

All participants had a colposcopic examination with the application of acetic acid to the cervix. Subsequently, participants in the EIS cohort underwent a ZedScan examination. ZedScan readings were made from 10 to 12 points clockwise around the cervix. On the Zedscan reading, red colour points out the area with the highest probability of high-grade disease, amber colour indicates possible high-grade areas and the absence of high-grade disease is indicated with green colour. In most women, 12 measurements cover well the junction area of the cervix. However, it might be possible that minor areas are omitted in case of very large cervix. After routine measurements (10-12 around the cervix) in case of suspected presence of CIN2+ by ZedScan, a particular single point mode can be used to localise more carefully the most abnormal area to be biopsied. In the EIS cohort, cervical biopsy sites were determined by the colposcopist based on both ZedScan results and colposcopic impression. The most severe histological diagnosis of all biopsies was recorded.

Random biopsies were not routinely taken in either of the cohorts. Colposcopy examination in both cohorts was based on Finnish Current Care Guidelines.[22] Five percent acetic acid and Lugol's iodine were available at the discretion of individual colposcopist to assess the abnormal cervical areas for biopsy. The colposcopic impression was recorded as high-grade, low-grade, or normal. Immediate LLETZ at initial visit ('select and treat'-approach) was performed when evaluated necessary according to Finnish Current Care

For peer review only - http://bmjopen.bmj.com/site/about/guidelines.xhtml

Guidelines: HSIL referral cytology with a colposcopic impression of CIN2+ entitled to perform LLETZ at the initial colposcopy with consent from the patient.[22] After cervical cytology with glandular atypia favouring neoplasia (AGC-FN) the Finnish Current Care Guidelines recommends immediate LLETZ irrespective of the age of the referred woman.[22]

Data analysis

We compared the prevalence of histologically confirmed CIN2+ lesions between the EIS and reference cohorts and calculated the sensitivity, specificity, positive and negative predictive values for colposcopy in both cohorts for the detection of CIN2+ lesions, both overall and stratified according to the referral cervical cytology. The positive test result for EIS cohort was defined as suspected presence of CIN2+ either by ZedScan and/or via colposcopic inspection. The test result was negative if both the colposcopic impression and ZedScan agreed on low-grade lesion or normal cervical finding, i.e. absence of CIN2+ lesion. In the reference cohort, positive test result was defined as a colposcopic impression of CIN2+ while negative test result was defined as the absence of changes suggesting CIN2+ lesions. The most advanced histopathological result of the biopsies or LLETZ specimen taken at the initial visit were used as a reference standard in both cohorts. Women without biopsies and with negative ZedScan result and normal colposcopic impression as well as low-grade referral were considered true negatives. Even though colposcopy and EIS examination were not truly independent tests in the setting used, we still performed a sensitivity analysis within the EIS cohort and assessed separately diagnostic testing accuracy of colposcopy and EIS in that cohort alone as well.

Risk ratio and risk difference were used to compare the sensitivity and specificity between the EIS and reference cohorts. The *p*-values <0.05 were considered statistically significant. All statistical analyses were performed using STATA/SE 15 (StataCorp, College Station TX, USA) and all statistical tests used were two-sided.

Patient and public involvement

For peer review only

For peer review only - http://bmjopen.bmj.com/site/about/guidelines.xhtml

Patients and/or the public were not involved in the design, conduct, reporting, or dissemination of this research.

Results

There were 1027 eligible women with adequate colposcopy and Zedscan examination performed In the EIS cohort. Altogether 68 women with other referral reasons than abnormal cervical cytology, 215 women with follow-up colposcopy visits and 97 women with missing data were excluded. In total, 647 women with new colposcopy referrals of abnormal cytology were included in the analysis (Figure S1, Table 1). Of all ZedScan procedures 75% were conducted by three individual colposcopists. The reference cohort included 1383 eligible women. Of these, 86 women were excluded due to other referral reasons than abnormal cervical cytology, 174 for having TZ3, 143 for missing relevant clinical data and 18 for pregnancy. As a result, a total of 962 women fulfilled the inclusion criteria (Figure S1, Table 1).

At least one biopsy was taken or imminent LLETZ made in 625 (96.6%) women in the EIS cohort and 952 (99.0%) in the reference cohort. Only one biopsy was taken from one quarter of women 165 (25.5%) in the EIS cohort and among 109 (11.3%) in the reference cohort, whereas twenty-two (3.4%) women in the EIS cohort and 10 (1.0%) in the reference cohort had no biopsy. The average number of biopsies was 1.8 if at least one biopsy was taken in the EIS cohort and 2.3 in the reference cohort (Table 1).

Altogether 222 (34.3%) women in the EIS cohort had CIN2+, including 5 (0.8%) cervical carcinomas and 14 (2.2%) adenocarcinoma in situ cases. In the reference cohort 391 (40.6%) women had CIN2+, including 7 (0.7%) cervical carcinomas and 15 (1.6%) adenocarcinoma in situ cases. (Table 1). The prevalence of CIN2+ was higher in the reference cohort among those referred for LSIL or ASC-H cytology, whereas the prevalence of CIN2+ was higher in the EIS cohort after ASC-US and HSIL referral cytology (Table 2, Table S1).

In the EIS cohort the overall sensitivity to detect CIN2+ was 94% (95% CI 90-97%) with corresponding specificity of 34% (95% CI 29-39%) (Table 2, Table S1). The sensitivity varied according to referral cytology, being the lowest, 77%, for LSIL cytology (95% CI 61-89%) and the highest for HSIL cytology with 100% sensitivity (95% CI 95-100%) (Table 2, Table S1). The specificity was lowest for HSIL cytology, 6% (95% CI 0-

For peer review only

For peer review only - http://bmjopen.bmj.com/site/about/guidelines.xhtml

Table 1. Characteristics of the electrical impedance spectroscopy (EIS) cohort and the reference cohort.

	EIS cohort		Reference cohort	
	n=647	%	n=962	%
Mean age	35.7		35.4	
Std.dev., Range	9.3 (20.3-76.4)		9.6 (19.2-67.8)	
**Age**				
<30 y	175	27.1	295	30.7
30-44 y	366	56.6	495	51.5
≥45 y	106	16.4	172	17.9
	647	100.0	962	100.0
**Referral cervical cytology stratified by age**				
ASC-US				
<30 y	28	4.3	43	4.5
30-44 y	52	8.0	28	2.9
≥45 y	14	2.2	28	2.9
LSIL				
<30 y	39	6.0	79	8.2
30-44 y	153	23.6	224	23.3
≥45 y	44	6.8	78	8.1
ASC-H				
<30 y	72	11.1	90	9.4
30-44 y	90	13.9	120	12.5
≥45 y	30	4.6	27	2.8
HSIL				
<30 y	31	4.8	75	7.8
30-44 y	54	8.3	102	10.6
≥45 y	9	1.4	23	2.4
AGC-NOS				
<30 y	5	0.8	5	0.5
30-44 y	15	2.3	12	1.2
≥45 y	8	1.2	11	1.1
AGC-FN				
<30 y	0	0.0	3	0.3
30-44 y	2	0.3	9	0.9
≥45 y	1	0.2	5	0.5
	647	100.0	962	100.0
**TZ type**				
TZ type 1	446	68.9	620	64.4
TZ type 2	201	31.1	342	35.6
	647	100.0	962	100.0
**Biopsies and LLETZ**				
No biopsy	22	3.4	10	1.0
1 biopsy	165	25.5	109	11.3
2 biopsies	263	40.6	420	43.7
3 biopsies	83	12.8	257	26.7
4 biopsies	1	0.2	43	4.5
5 biopsies	0	0.0	5	0.5
LLETZ	113	17.5	118	12.3
	647	100.0	962	100.0
**Histology**				
No biopsy	22	3.4	10	1.0
Normal histology	222	34.3	247	25.7
CIN1 (LSIL)	181	28.0	312	32.4
CIN2 (HSIL)	95	14.7	210	21.8
CIN3 (HSIL)	107	16.5	154	16.0
Glandular atypia	1	0.2	7	0.7
AIS	14	2.2	15	1.6
Adenocarcinoma	3	0.5	3	0.3
Sq. cell carcinoma	2	0.3	4	0.4
	647	100.0	962	100.0

Std. dev.: standard deviation; EIS: electrical impedance spectroscopy; CIN: cervical intraepithelial neoplasia; TZ: transformation zone; ASC-US: atypical squamous cells of undetermined significance; LSIL: low-grade squamous intraepithelial lesion; HSIL: high-grade squamous intraepithelial lesion; ASC-H: atypical squamous cells that cannot exclude HSIL; AGC-NOS: atypical glandular cells not otherwise specified; AGC-FN: atypical glandular cells that favor neoplasia; AIS: adenocarcinoma in situ; sq. cell carcinoma: squamous cell carcinoma; LLETZ: large loop excision of the transformation zone

For peer review only

For peer review only - http://bmjopen.bmj.com/site/about/guidelines.xhtml

Table 2. Sensitivity, specificity of the electrical impedance spectroscopy (EIS) cohort and the reference cohort for the detection of CIN2+ lesions by cervical cytology, TZ type and age group.

	EIS cohort (n=647)				Refence cohort (n=962)			
	CIN2+/n	Colpo+ZS CIN2+[1]	Sensitivity	Specificity	CIN2+/n	Colpo CIN2+[2]	Sensitivity	Specificity
ASC-US	16/94	15	94(70-100)	47(36-59)	9/99	5	56(21-86)	97(91-99)
LSIL	39/236	30	77(61-89)	42(35-49)	72/381	31	43(31-55)	92(89-95)
ASC-H	85/192	84	99(94-100)	11(6-19)	138/237	87	63(54-71)	65(54-74)
HSIL	77/94	77	100(95-100)	6(0-29)	154/200	133	86(80-91)	46(31-61)
AGC-NOS	3/28	2	67(9-99)	44(24-65)	5/28	3	60(15-95)	96(78-100)
AGC-FN	2/3	1	50(1-99)	100(3-100)	13/17	8	62(32-86)	25(1-81)
ALL	222/647	209	94(90-97)	34(29-39)	391/962	267	68(63-73)	84(81-87)
TZ1	156/446	146	94(89-97)	31(26-37)	279/620	187	67(61-73)	84(80-88)
TZ2	66/201	63	96(87-99)	40(32-49)	112/342	80	71(62-80)	84(79-88)
<30 y	60/175	56	93(84-98)	35(26-44)	134/295	96	72(63-79)	77(70-83)
30-44 y	131/366	124	95(89-98)	33(27-40)	211/495	144	68(62-75)	86(81-90)
>45 y	31/106	29	94(79-99)	35(24-47)	46/172	27	59(43-73)	89(82-94)
HG cytology	164/289	162	99(96-100)	11(6-18)	305/454	228	75(70-80)	58(49-66)
LG cytology	58/358	47	81(69-90)	43(38-49)	86/508	39	45(35-57)	93(91-96)
ASC-H, HSIL	162/286	161	99(97-100)	11(6-17)	292/437	220	75(70-80)	59(50-67)
ASC-US,LSIL	55/330	45	82(69-91)	43(37-49)	81/480	36	44(33-56)	93(90-96)
Glandular	5/31	3	60(15-95)	46(27-67)	18/45	11	61(36-83)	85(66-96)
1 biopsy	11/165	7	64(31-89)	51(43-59)	14/109	5	36(13-65)	99(94-100)
2 biopsies	78/263	70	90(81-96)	23(17-30)	112/420	66	59(49-68)	90(86-93)
≥3 biopsies	43/84	43	100	0	168/305	113	67(60-74)	67(59-75)
LLETZ	90/113	89	99(94-100)	4(0-22)	97/118	83	86(77-92)	38(18-62)

EIS: electrical impedance spectroscopy; CIN: cervical intraepithelial neoplasia; TZ: transformation zone; ASC-US: atypical squamous cells of undetermined significance; LSIL: low-grade squamous intraepithelial lesion; HSIL: high-grade squamous intraepithelial lesion; ASC-H: atypical squamous cells that cannot exclude HSIL; AGC-NOS: atypical glandular cells not otherwise specified; AGC-FN: atypical glandular cells that favor neoplasia; LLETZ: large loop excision of the transformation zone; HG high grade; LG: low grade

[1]Colposcopic impression and/or ZedScan result of CIN2+ of histologically confirmed CIN2+ cases.

[2]Colposcopic impression of CIN2+ of histologically confirmed CIN2+ cases.

29%), and highest for ASC-US, 47% (95% CI 36-59%). EIS missed 3 low-grade referral cases of CIN2+ identified by the colposcopist (two cases if CIN2 and one CIN3). Colposcopic impression was less than CIN2 in 43 CIN2+ cases that were detected by ZedScan. A total of 13 cases (5.9%) of CIN2+ were missed by both ZedScan and the colposcopist (biopsies were still taken due to suspicion of low-grade lesion), including two adenocarcinoma in situ cases and eleven high-grade lesions (nine CIN2 and two CIN3 cases).

In the reference cohort, the overall sensitivity to detect CIN2+ was 68% (95% CI 63-73%) with corresponding specificity of 84% (95% CI 81-87%) (Table 2, Table S1). The sensitivity to detect CIN2+ by colposcopic impression of CIN2+ was the lowest after LSIL cytology, 43%, and the highest after HSIL cytology, 86% (Figure 1, Table 2, Table S1). Overall, the colposcopic impression was less than CIN2+ in 31.7% (124/391) of CIN2+ cases and biopsies were taken due to suspicion of a low-grade lesion. Results

For peer review only

For peer review only - http://bmjopen.bmj.com/site/about/guidelines.xhtml

stratified according to TZ type, age, and referral cytology are presented in Table S2. There was no obvious impact of age on specificity or sensitivity within different cytologies (Table S2).

Compared to the referral cohort, the sensitivity to detect CIN2+ was higher in the EIS cohort overall, with risk ratio (RR) of 1.38 (95% CI 1.28-1.49), and after LSIL, ASC-H and HSIL referral cervical cytologies (Table 3, Table S3). TZ 1 and taking two or more biopsies were associated with higher observed sensitivity (Table 3, Table S3). Specificity was correspondingly lower in the EIS cohort overall as well as when stratified according to referral cytology (Table 3, Table S3).

Table 3. Sensitivity and specificity of the electrical impedance spectroscopy cohort (EIS) and the reference cohort by cytology, TZ type and age group in identifying CIN2+, with corresponding risk ratios (RR) of sensitivity and specificity.

	EIS Sensitivity	Reference Sensitivity	Sensitivity RR (95%)[1]	p.	EIS Specificity	Reference Specificity	Specificity RR (95%)[1]	p.
ASC-US	94(70-100)	56(21-86)	1.69(0.93-3.07)	0.0219	47(36-59)	97(91-99)	0.49(0.39-0.62)	<0.0001
LSIL	77(61-89)	43(31-55)	1.79(1.30-2.45)	0.0006	42(35-49)	92(89-95)	0.45(0.38-0.53)	<0.0001
ASC-H	99(94-100)	63(54-71)	1.57(1.38-1.78)	<0.0001	11(6-19)	65(54-74)	0.17(0.10-0.30)	<0.0001
HSIL	100(95-100)	86(80-91)	1.16(1.09-1.23)	0.0007	6(0-29)	46(31-61)	0.13(0.02-0.89)	0.0033
AGC-NOS	67(9-99)	60(15-95)	1.11(0.38-3.25)	0.8504	44(24-65)	96(78-100)	0.46(0.29-0.72)	0.0001
AGC-FN	50(1-99)	62(32-86)	0.81(0.19-3.47)	0.7565	100(3-100)	25(1-81)	4.0(0.73-21.84)	0.1709
ALL	94(90-97)	68(63-73)	1.38(1.28-1.49)	<0.0001	34(29-39)	84(81-87)	0.40(0.35-0.46)	<0.0001
TZ1	94(89-97)	67(61-73)	1.40(1.27-1.53)	<0.0001	31(26-37)	84(80-88)	0.37(0.31-0.44)	<0.0001
TZ2	96(87-99)	71(62-80)	1.34(1.18-1.57)	0.0001	40(32-49)	84(79-88)	0.48(0.38-0.59)	<0.0001
<30 y	93(84-98)	72(63-79)	1.30(1.15-1.48)	0.0007	35(26-44)	77(70-83)	0.45(0.35-0.59)	<0.0001
30-44 y	95(89-98)	68(62-75)	1.39(1.25-1.53)	<0.0001	33(27-40)	86(81-90)	0.39(0.32-0.47)	<0.0001
≥45 y	94(79-99)	59(43-73)	1.59(1.23-2.07)	0.0008	35(24-47)	89(82-94)	0.39(0.28-0.54)	<0.0001
HG cytology	99(96-100)	75(70-80)	1.32(1.24-1.41)	<0.0001	11(6-18)	58(49-66)	0.19(0.12-0.32)	<0.0001
LG cytology	81(69-90)	45(35-57)	1.79(1.37-2.33)	<0.0001	43(38-49)	93(91-96)	0.46(0.41-0.53)	<0.0001
1 biopsy	64(31-89)	36(13-65)	1.78(0.77-4.10)	0.1654	51(43-59)	99(94-100)	0.51(0.44-0.60)	<0.0001
2 biopsies	90(81-96)	59(49-68)	1.52(1.28-1.81)	<0.0001	23(17-30)	90(86-93)	0.26(0.20-0.34)	<0.0001
≥3 biopsies	100	67(60-74)	1.49(1.34-1.65)	<0.0001	0	67(59-75)	0	<0.0001

EIS: electrical impedance spectroscopy; CIN: cervical intraepithelial neoplasia; TZ: transformation zone; ASC-US: atypical squamous cells of undetermined significance; LSIL: low-grade squamous intraepithelial lesion; HSIL: high-grade squamous intraepithelial lesion; ASC-H: atypical squamous cells that cannot exclude HSIL; AGC-NOS: atypical glandular cells not otherwise specified; AGC-FN: atypical glandular cells that favor neoplasia; HG: high grade; LG: low grade
[1]The values of risk ratio >1 imply better/improved effect with ZedScan

For peer review only - http://bmjopen.bmj.com/site/about/guidelines.xhtml

In the EIS cohort, colposcopic impression of high-grade disease (CIN2+) was present with EIS indicating the presence of CIN2+ in 73.4% of all histologically confirmed CIN2+ cases. In the sensitivity analysis within the EIS cohort, colposcopy alone was indicative for the presence of CIN2+ in 166 of 222 CIN2+ cases (74.8%) and ZedScan in 206 of 222 (92.8%) of CIN2+ cases, suggesting an additional 40 cases (24.1%) detected by ZedScan only. The additional cases increased the detection of CIN2+ from 30 to 44 in women with low-grade cytology and from 136 to 162 in women with high-grade cytology (Figure 1). The sensitivity to detect CIN2+ by colposcopy alone according to referral cytology was otherwise similar between the cohorts, except for women with ASC-H cervical cytology the colposcopy alone in the EIS cohort seemed to detect more CIN2+ cases (p=0.02) (Figure 1). Among colposcopists who performed colposcopies in both cohorts, the average number of biopsies by cytology were higher in all cytology groups in the reference cohort compared to the EIS cohort. The average number of biopsies varied between 1.7-2.3 in the EIS cohort and between 2.2-2.8 in the reference cohort (Table S4).

Discussion

We compared the performance of colposcopy in detecting CIN2+ according to referral cervical cytology with and without EIS as an adjunctive technology. Colposcopy combined with EIS seemed to have a higher sensitivity, but a lower specificity compared to conventional colposcopy, regardless of the referral cervical cytology. The prevalence of CIN2+ lesions was higher in the EIS cohort after ASC-US and HSIL referral, but lower after LSIL and ASC-H cervical cytology. The average number of biopsies was lower in the EIS cohort.

Overall, EIS performed well with a high sensitivity (94%) but had a low specificity (34%) consistent with the previous studies.[13, 14, 16] Here, the sensitivity might have been overestimated in both cohorts as the true positive result was based on histology data at first visit only and lesions missed at first visit and detected during the follow-up were not included in either cohort. Still, this would not affect the estimates of relative performance. The sensitivity (68%) and specificity (84%) of colposcopy in the reference cohort was as well in line with existing data.[5, 7, 23]

For peer review only

For peer review only - http://bmjopen.bmj.com/site/about/guidelines.xhtml

The increased detection of CIN2+ cases by EIS has been reported as most pronounced in women with low-grade cytology [13, 14, 16, 17] or with high-risk HPV positivity without cytological changes.[16, 18] In our study, additional cases of CIN2+ detected by EIS were also most frequent among low-grade referrals. Furthermore, the sensitivity to detect CIN2+ with EIS was higher in most cervical cytology groups (ASC-US, LSIL, ASC-H, HSIL) compared to colposcopy alone. Only within HSIL cytology EIS combined with colposcopy detected all CIN2+ cases. In women with other referral cytology (ASC-US, LSIL, ASC-H) there were cases of CIN2+ that EIS did not detect, but where biopsy of CIN2+ was warranted based on colposcopic diagnosis. Nevertheless, missed cases of CIN2+ were even more frequent in the reference cohort, where more CIN2+ lesions were detected in biopsies with colposcopic impression of CIN1 or lower. Contrary to expectations, the prevalence of CIN2+ was higher in the EIS cohort only after ASC-US and HSIL referral cytology. One explanation for lower prevalence of CIN2+ lesions in the EIS cohort after LSIL and ASC-H cytology could be that routine practice in Finland is to take biopsies also from low-grade lesions, rather than to abstain from taking biopsies when CIN2+ lesions are not colposcopically suspected. Biopsies even from mild acetowhite lesions are important in excluding a high-grade disease as the sensitivity of colposcopy to detect CIN2+ is far from 100%. Such biopsies could well have been more frequent without than with EIS as an additional confirmation on suspected absence of CIN2+. This is supported by the observation that two or more biopsies were taken from 54% of women in the EIS cohort, whereas up to 75% of women in the reference cohort had at least two biopsies. In addition, the average number of biopsies by cytology among colposcopists who performed colposcopies in both cohorts were constantly higher in the reference cohort compared to the EIS cohort reflecting a change in manner/threshold to take biopsies when ZedScan was used as an adjunct technology. Multiple biopsies are known to increase the sensitivity of colposcopy as at least small lesions can easily be missed.[24, 25] In women with low-grade referral cervical cytology, a single biopsy has shown to be insufficient to rule out a high-grade disease.[26] A British survey has also reported experienced colposcopists to take mostly two biopsies in diagnosing high-grade disease.[27] A Danish study found taking four biopsies to increases the detection rate of cervical dysplasia to 95.2%.[28] The average

For peer review only - http://bmjopen.bmj.com/site/about/guidelines.xhtml

number of biopsies in the EIS cohort was higher (1.84) compared to previous reports (1.07 and 1.51),[13, 14] but still lower than in the reference cohort (2.3).

Our observation of overall fewer biopsies along with fewer CIN2+ lesions detected in the EIS cohort can either indicate a true difference in CIN2+ prevalence between the cohorts, selection bias towards using EIS preferably on patients in whom CIN2+ lesion is not clearly present, or that CIN2+ lesions could have been missed in the EIS cohort, especially after LSIL and ASC-H referral cytology. If lesions were missed, it could possibly be due to a higher biopsy threshold in the EIS cohort, as indicated by lower number of biopsies. Without longitudinal data we still cannot be certain whether prevalent CIN2+ cases were indeed more frequently missed at the first visit in the EIS cohort. The prevalence of CIN2+ in EIS cohort in women with high-grade cytology (ASC-H and HSIL) is below previous observations (56.7% vs. 79.1-84.0%).[13, 16] However, when restricted to only women with HSIL referral cervical cytology or low-grade (ASC-US and LSIL) cytology, the prevalence for CIN2+ here did not differ from previous reports.[13, 16] Cytological diagnoses may well vary between cytopathologists as well as between countries and this possible difference in classification might also explain the observed difference in CIN2+ prevalence, especially after ASC-H cytology.[29] The longitudinal data on EIS results are scarce. In women referred with low-grade cytology, the future risk of CIN2+ was increased in up to 36 months follow-up if both colposcopic impression and EIS results were indicative for CIN2+ compared with women with other combinations of these two parameters, suggesting that EIS might provide new information on the future risk of high-grade disease.[30]

Strengths and limitations

Most previous studies have compared the performance of EIS as an adjunctive technology for colposcopy against conventional colposcopy within the cohort where EIS was used, even though in clinical setting EIS is not a truly independent measurement from colposcopy. To our knowledge this is the first report on the performance of EIS as an adjunctive technology for colposcopy stratified according to referral cytology and compared to an external reference cohort. Even though our cohorts were collected at different time

For peer review only - http://bmjopen.bmj.com/site/about/guidelines.xhtml

periods, they both represent women in the same catchment area referred to colposcopy due to abnormal cervical cytology. All colposcopies were performed in the same clinic by experienced colposcopists. Furthermore, none of the authors of this work have financial conflicts of interest with the technology studied. Our study also has some limitations. It is not possible to rule out that there would not have been any variation in sensitivity or specificity between the cohorts in different time periods. EIS device is not truly independent of colposcpic skills and the colposcopic performance can vary depending on the colposcopist. Also, the referral cytology and the colposcopic impression are incorporated in the EIS analysis by ZedScan. In order to take into account the variation of colposcopic performance and reliance on EIS device we collected a large cohort representing routine work. Including colposcopic examinations by several different colposcopists represents a real-life situation which could be considered as a strength compared to studies where all colposcopies have been performed by a single colposcopist.

When the cervical transformation zone is not fully visible, TZ type 3, ZedScan technology cannot be reliably applied and the results are not applicable to this population. CIN2+ lesions could well have been missed in both cohorts since the results are based on data collected on the initial visit. EIS might miss some lesions that either could have been detected with lower biopsy threshold or where biopsy would not have been indicated even in conventional colposcopy. However, complete certainty of the histology would have required LLETZ for all participants which would not have been ethically just.

Conclusions

Colposcopy with EIS has a higher sensitivity and a lower specificity in identifying CIN2+ compared to conventional colposcopy, irrespective of cervical cytology. EIS can, therefore, be assumed to be of clinical benefit in colposcopy, particularly in women with low-grade cervical cytology where the prevalence of CIN2+ is low. We also observed an overall lower prevalence of CIN2+ lesions in the EIS cohort compared to a reference cohort with conventional colposcopy. The performance of EIS as an adjunctive technology for colposcopy has not been previously compared by cytology to an external reference cohort. While the observation of lower CIN2+ rate could be explained by different CIN2+ prevalence between the cohorts or

For peer review only - http://bmjopen.bmj.com/site/about/guidelines.xhtml

selection bias, the finding is important and warrants further research, especially along with the observed lower number of biopsies in the EIS cohort. Adjunctive technologies are likely to become increasingly appealing in colposcopy, as the prevalence of high-grade cervical lesions is declining. Randomised controlled trials comparing EIS with a conventional colposcopy, including women referred due to persistent HPV infection without cytological changes are warranted. Before such further evidence, firm recommendations on applicability of EIS as an adjunctive technology for colposcopy cannot be made.

Figure 1. Numbers and rates of CIN2+ lesions detected in the electrical impedance spectroscopy cohort (EIS) and in the reference cohort according to referral cytology. (A) Numbers and rates of CIN2+ detected by ZedScan alone and reference cohort stratified according to referral cytology (B) Numbers and rates of CIN2+ detected by colposcopy alone in EIS and reference cohorts stratified according to referral cytology. Numbers of patients are given in the columns.

For peer review only

Contributors

PN and IK were responsible for the conceptualisation and design of the study as well as methodology. XC, KL, LKT contributed to conceptualisation. LB performed the statistical analysis with the aid of IK. LB, PN, MK, PLO, SV and AH were responsible for data collection. LB drafted the original manuscript and IK, PN, CR, XC, KL, LKT, AH and KA participated in writing, reviewing and editing. All authors listed qualify for authorship and approved the final version of the paper.

Funding

This work was supported by the Academy of Finland (IK), the Finnish Medical Foundation (IK), and Finnish State Research Funding (IK).

Competing interests None declared.

Patient consent for publication Not required.

Ethics approval This study and data collection on patients where EIS was used was considered as a service evaluation and therefore a separate ethical approval was not required as per consultation with Helsinki-Uusimaa Hospital District Ethical Committee. For the historical reference cohort an ethical approval was received from Helsinki-Uusimaa Hospital District Ethical Committee (ref. no. 130/13/03/03/2013).

Data availability statement Data are available upon reasonable request.

For peer review only

For peer review only - http://bmjopen.bmj.com/site/about/guidelines.xhtml

References:

1 Munro A, Gillespie C, Cotton S, et al. The impact of human papillomavirus type on colposcopy performance in women offered HPV immunisation in a catch-up vaccine programme: a two-centre observational study. *BJOG* 2017;124(9):1394–401.

2 Lehtinen M, Pimenoff VN, Nedjai B, et al. Assessing the risk of cervical neoplasia in the post-HPV vaccination era. *Int J Cancer* 2022;(June):1–9.

3 Rebolj M, Rimmer J, Denton K, et al. Primary cervical screening with high risk human papillomavirus testing: Observational study. *BMJ* 2019;364:l240.

4 Zuchna C, Hager M, Tringler B, et al. Diagnostic accuracy of guided cervical biopsies: A prospective multicenter study comparing the histopathology of simultaneous biopsy and cone specimen. *Am J Obstet Gynecol* 2010;203(4):321.e1-321.e6. Available: http://dx.doi.org/10.1016/j.ajog.2010.05.033

5 Cantor SB, Cárdenas-Turanzas M, Cox DD, et al. Accuracy of colposcopy in the diagnostic setting compared with the screening setting. *Obstet Gynecol* 2008;111(1):7–14.

6 Underwood M, Arbyn M, Parry-Smith W, et al. Accuracy of colposcopy-directed punch biopsies: a systematic review and meta-analysis. *BJOG* 2012;119(11):1293–1301.

7 Brown BH, Tidy JA. The diagnostic accuracy of colposcopy – A review of research methodology and impact on the outcomes of quality assurance. *Eur J of Obstet Gynecol Repro Biol* 2019;240(2019):182–6. Available: https://doi.org/10.1016/j.ejogrb.2019.07.003

8 Demarco M, Lorey TS, Fetterman B, et al. Risks of CIN 2 +, CIN 3 +, and Cancer by Cytology and Human Papillomavirus Status: The Foundation of Risk-Based Cervical Screening Guidelines. *J Low Genit Tract Dis* 2017;21(4):261–7.

9 Zilico Ltd. Manchester: Available: https://zilico.co.uk

10 Tidy JA, Brown BH, Healey TJ, et al. Accuracy of detection of high-grade cervical intraepithelial neoplasia using electrical impedance spectroscopy with colposcopy. *BJOG* 2013;120(4):400–11.

For peer review only

For peer review only - http://bmjopen.bmj.com/site/about/guidelines.xhtml

11 Brown BH, Tidy JA, Boston K, et al. Relation between tissue structure and imposed electrical current flow in cervical neoplasia. *The Lancet* 2000 Mar 11;355(9207):892–5. Available: https://doi.org/10.1016/S0140-6736(99)09095-9

12 Balasubramani L, Brown BH, Healey J, et al. The detection of cervical intraepithelial neoplasia by electrical impedance spectroscopy: The effects of acetic acid and tissue homogeneity. *Gynecol Oncol* 2009;115(2):267–71. Available: http://dx.doi.org/10.1016/j.ygyno.2009.08.010

13 Tidy JA, Brown BH, Lyon RE, et al. Are colposcopy and electrical impedance spectroscopy complementary when used to detect high-grade cervical neoplasia? *Eur J Gynaecol Oncol* 2018;39(1):70–5.

14 Muszynski C, Dupont E, Vaysse B, et al. The impact of using electrical impedance spectroscopy (ZedScan) on the performance of colposcopy in diagnosing high grade squamous lesions of the cervix. *J Gynecol Obstet Hum Reprod* 2017;46(9):669–73. Available: http://dx.doi.org/10.1016/j.jogoh.2017.08.007

15 Homola W, Fuchs T, Baranski P, et al. Use of electrical impedance spectroscopy as an adjunct to colposcopy in a pathway of cervical intraepithelial neoplasia diagnostics. *Ginekol Pol* 2019;90(11):628–32.

16 Tidy JA, Brown BH. Increased detection of high grade CIN, when using electrical impedance spectroscopy as an adjunct to routine colposcopy, is maintained when used across international boundaries: Prospective data from nine European countries. *Eur J Obstet and Gynecol Repro Biol* 2022;275(May):41–5. Available: https://doi.org/10.1016/j.ejogrb.2022.05.025

17 Tsampazis N, Vavoulidis E, Siarkou CM, et al. Diagnostic comparison of electrical impedance spectroscopy with colposcopy and HPV mRNA-testing in the prediction of CIN2+ women in Greece. *J Obstet Gynaecol Res* 2023 Apr;49(4):1222-1229. doi: 10.1111/jog.15557. Epub 2023 Jan 19. PMID: 36658620.

For peer review only - http://bmjopen.bmj.com/site/about/guidelines.xhtml

439 18 Tidy JA, Lyon R, Ellis K, et al. The impact of age and high-risk human papillomavirus (hrHPV) status on the prevalence of high-grade cervical intraepithelial neoplasia (CIN2+) in women with persistent hrHPV-positive, cytology-negative screening samples: a prospective cohort study. *BJOG* 2020;127(10):1260–7.

442 19 Macdonald M, Brown B, Lyon R, et al. Influence of high risk HPV genotype on colposcopic performance: A large prospective study demonstrates improved detection of disease with ZedScan I, particularly in non-HPV 16 patients*. Eur J Obstet Gynecol Reprod Biol* 2017 Apr;211:194-198. doi: 10.1016/j.ejogrb.2017.02.020. Epub 2017 Feb 20. PMID: 28292693.

446 20 National Institute for Health and Care Excellence. NICE Adjunctive colposcopy technologies for assessing suspected cervical abnormalities: the DYSIS colposcope with DYSISmap and the Zedscan I. 2018. Available: https://www.nice.org.uk/guidance/dg32 [Accessed 22 Jan 2022].

449 21 Aro K, Nieminen P, Louvanto K, et al. Gynecologic Oncology Age-specific HPV type distribution in high-grade cervical disease in screened and unvaccinated women. *Gynecol Oncol* 2019;154(2):354–9. Available: https://doi.org/10.1016/j.ygyno.2019.05.024

452 22 Working group set up by the Finnish Medical Society Duodecim and the Finnish Colposcopy Association. Cytological Changes in the Cervix, Vagina and Vulva. Current Care Guidelines. The Finnish Medical Society Duodecim, 2021. Available: www.kaypahoito.fi [Accessed 17 Jan 2022].

455 23 van der Marel J, van Baars R, Quint WGV, et al. The impact of human papillomavirus genotype on colposcopic appearance: A cross-sectional analysis. *BJOG* 2014;121(9):1117–26.

457 24 Pretorius RG, Belinson JL, Burchette RJ, et al. Regardless of skill, performing more biopsies increases the sensitivity of colposcopy. *J Low Genit Tract Dis* 2011;15(3):180–8.

459 25 Wentzensen N, Walker JL, Gold MA, et al. Multiple biopsies and detection of cervical cancer precursors at colposcopy. *J Clin Oncol* 2015;33(1):83–9.

461 26 Moss EL, Hadden P, Douce G, et al. Is the colposcopically directed punch biopsy a reliable diagnostic test in women with minor cytological lesions? *J Low Genit Tract Dis* 2012;16(4):421–6.

For peer review only - http://bmjopen.bmj.com/site/about/guidelines.xhtml

27 Myriokefalitaki E, Redman CWE, Potdar N, et al. The Use of the Colposcopically Directed Punch Biopsy in Clinical Practice: A Survey of British Society of Colposcopy and Cervical Pathology (BSCCP)-Accredited Colposcopists. *J Low Genit Tract Dis* 2016;20(3):234–8.

28 Booth B, Petersen L, Blaakaer J, et al. Accuracy of colposcopy-directed biopsy vs dynamic spectral imaging directed biopsy in correctly identifying the grade of cervical dysplasia in women undergoing conization: A methodological study. *Acta Obstet Gynecol Scand 2020* Aug;99(8):1064-1070. doi: 10.1111/aogs.13832. Epub 2020 Mar 9. PMID: 32105344.

29 Sherman ME, Castle PE, Solomon D. Cervical cytology of atypical squamous cells-cannot exclude high-grade squamous intraepithelial lesion (ASC-H): Characteristics and histologic outcomes. *Cancer* 2006 Oct 25;108(5):298–305.

30 Brown BH, Highfield PE, Tidy JA. Prognostic value of electrical impedance spectroscopy (EIS) when used as an adjunct to colposcopy - A longitudinal study. *J Electr Bioimp* 2020;11(1):81–6.

For peer review only - http://bmjopen.bmj.com/site/about/guidelines.xhtml

(A) (B)

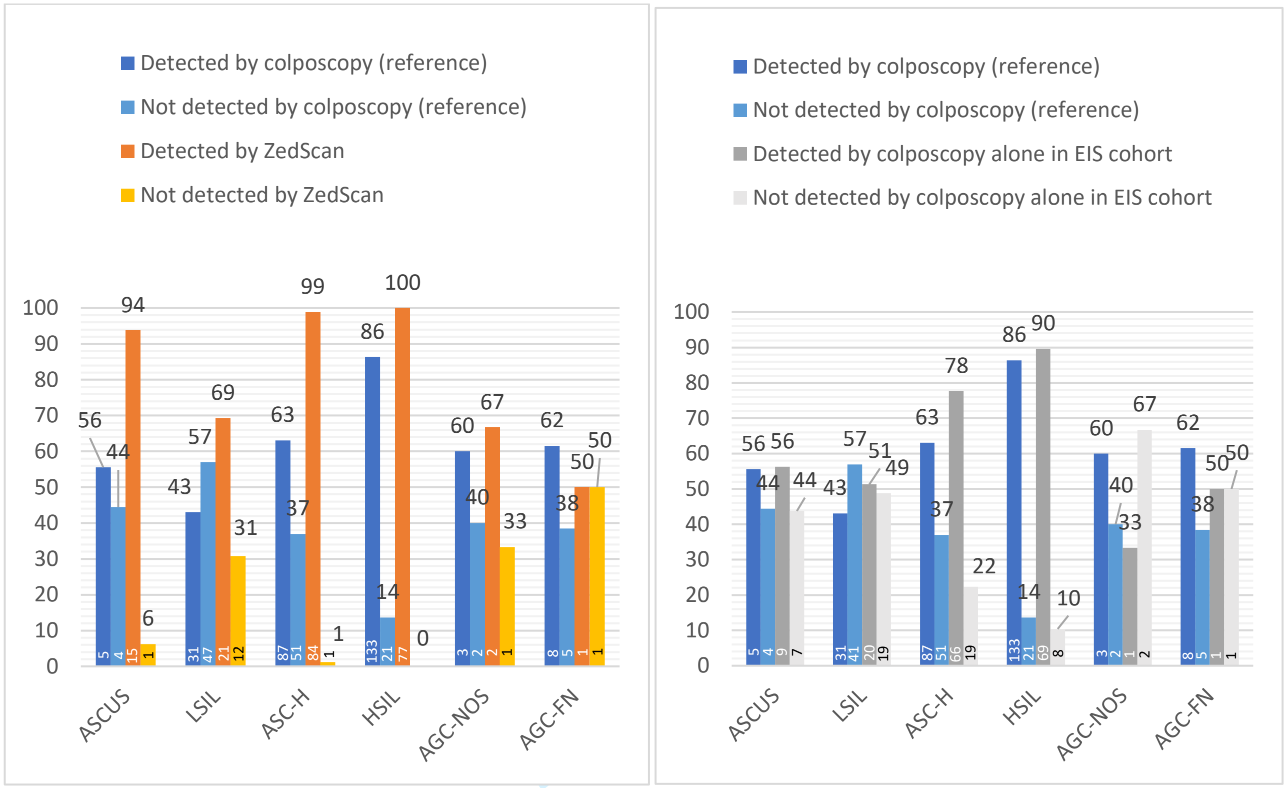

review only

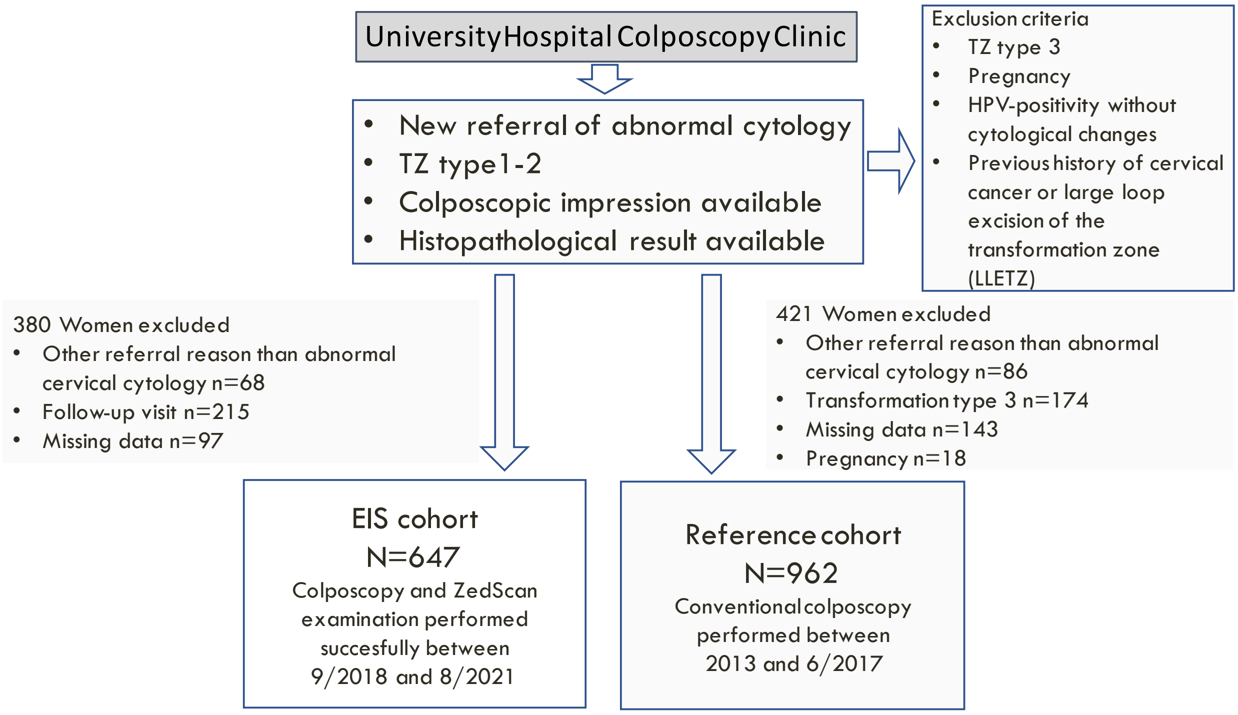

Figure S1. Flow-chart of the study comparing the performance of colposcopy by referral cervical cytology in two cohorts with and without electrical impedance spectroscopy as an adjunctive technology.

Table S1. Sensitivity, specificity, positive and negative predictive value of the electrical impedance spectroscopy (EIS) cohort and the reference cohort for the detection of CIN2+ lesions within different cervical cytology by TZ type and age group.

	EIS cohort (n=647)								Reference cohort (n=962)							
		Colpo+ZS[1]			Colpo+ZS[3]					Colpo[2]			Colpo[4]			
	CIN2+/n	CIN2+	Sensitivity	<CIN/n	<CIN2	Specificity	PPV	NPV	CIN2+/n	CIN2+	Sensitivity	<CIN2/n	<CIN2	Specificity	PPV	NPV
ASC-US	16/94	15	94(70-100)	78/94	37	47(36-59)	27(16-40)	97(86-100)	9/99	5	56(21-86)	90/99	87	97(91-99)	63(25-92)	96(89-99)
TZ1	11/66	10	91(59-100)	55/66	24	44(30-58)	24(12-40)	96(80-100)	7/57	3	43(10-82)	50/57	47	94(84-99)	50(12-88)	92(81-98)
TZ2	5/28	5	100(48-100)	23/28	13	57(35-77)	33(12-62)	100(75-100)	2/42	2	100(16-100)	40/42	40	100(91-100)	100(16-100)	100(91-100)
<30 y	6/28	5	83(36-100)	22/28	12	55(32-76)	33(12-62)	92(64-100)	1/43	0	0(0-98)	42/43	39	93(81-99)	0(0-71)	98(87-100)
30-44 y	10/52	10	100(69-100)	42/52	21	50(34-66)	32(17-51)	100(84-100)	7/28	4	57(18-90)	21/28	21	100(84-100)	100(40-100)	88(68-97)
>45 y	0/14	0	0	14/14	4	29	0	100	1/28	1	100(3-100)	27/28	27	100(87-100)	100(3-100)	100(87-100)
LSIL	39/236	30	77(61-89)	197/236	82	42(35-49)	21(14-28)	90(82-95)	72/381	31	43(31-55)	309/381	285	92(89-95)	56(42-70)	87(83-91)
TZ1	26/157	20	77(56-91)	131/157	53	41(32-49)	20(13-30)	90(79-96)	53/235	23	43(30-58)	182/235	169	93(88-96)	64(46-79)	85(79-90)
TZ2	13/79	10	77(46-95)	66/79	29	44(32-57)	21(11-36)	91(75-98)	19/146	8	42(20-67)	127/146	116	91(85-96)	42(20-67)	91(85-96)
<30 y	4/39	1	25(1-81)	35/39	21	60(42-76)	7(0-32)	88(68-97)	17/79	9	53(28-77)	62/79	54	87(76-94)	53(28-77)	87(76-94)
30-44 y	28/153	24	86(67-96)	125/153	46	37(28-46)	23(16-33)	92(81-98)	46/224	19	41(27-57)	178/224	166	93(89-97)	61(42-78)	86(80-91)
>45 y	7/44	5	71(29-96)	37/44	15	41(25-58)	19(6-38)	88(64-99)	9/78	3	33(8-70)	69/78	65	94(86-98)	43(10-82)	92(83-97)
ASC-H	85/192	84	99(94-100)	107/192	12	11(6-19)	47(39-55)	92(64-100)	138/237	87	63(54-71)	99/237	64	65(54-74)	71(62-79)	56(46-65)
TZ1	57/134	56	98(91-100)	77/134	6	8(3-16)	44(35-53)	86(42-100)	100/167	64	64(54-73)	67/167	42	63(50-74)	72(61-81)	54(42-65)
TZ2	28/58	28	100(88-100)	30/58	6	20(8-39)	54(40-68)	100(54-100)	38/70	23	61(43-76)	32/70	22	69(50-84)	70(51-84)	60(42-75)
<30 y	24/72	24	100(86-100)	48/72	5	10(4-23)	36(25-49)	100(48-100)	57/90	34	60(46-72)	33/90	18	55(36-72)	69(55-82)	44(29-60)
30-44 y	46/90	45	98(89-100)	44/90	4	9(3-22)	53(42-64)	80(28-100)	67/120	45	67(55-78)	53/120	37	70(56-82)	74(61-84)	63(49-75)
>45 y	15/30	15	100(78-100)	15/30	3	20(4-48)	56(35-75)	100(29-100)	14/27	8	57(29-82)	13/27	9	69(39-91)	67(35-90)	60(32-84)
HSIL	77/94	77	100(95-100)	17/94	1	6(0-29)	83(74-90)	100(3-100)	154/200	133	86(80-91)	46/200	21	46(31-61)	84(78-90)	50(34-66)
TZ1	58/67	58	100	9/67	0	0	87	0	104/131	88	85(76-91)	27/131	15	56(35-75)	88(80-94)	48(30-67)
TZ2	19/27	19	100(82-100)	8/27	1	13(0-53)	73(52-88)	100(3-100)	50/69	45	90(78-97)	19/69	6	32(13-57)	78(65-88)	55(23-83)
<30 y	25/31	25	100	6/31	0	0	81	0	54/75	48	89(77-96)	21/75	10	48(26-70)	81(69-90)	63(35-85)
30-44 y	45/54	45	100(92-100)	9/54	1	11(0-48)	85(72-93)	100(3-100)	84/102	72	86(76-92)	18/102	9	50(26-74)	89(80-95)	43(22-66)
>45 y	7/9	7	100	2/9	0	0	78	0	16/23	13	81(54-96)	7/23	2	29(4-71)	72(47-90)	40(5-85)

EIS: electrical impedance spectroscopy; CIN: cervical intraepithelial neoplasia; TZ: transformation zone; PPV: positive predictive value; NPV: negative predictive value; ASC-US: atypical squamous cells of undetermined significance; LSIL: low-grade squamous intraepithelial lesion; HSIL: high-grade squamous intraepithelial lesion; ASC-H: atypical squamous cells that cannot exclude HSIL; AGC-NOS: atypical glandular cells not otherwise specified; AGC-FN: atypical glandular cells that favor neoplasia; LLETZ: large loop excision of the transformation zone; HG: high grade; LG: low grade

[1]Colposcopic impression and/or ZedScan result of CIN2+ of histologically confirmed CIN2+ cases.

[2]Colposcopic impression of CIN2+ of histologically confirmed CIN2+ cases.

[3]Colposcopic impression and ZedScan result less than CIN2 of histologically confirmed cases <CIN2.

[4]Colposcopic impression less than CIN2 of histologically confirmed cases <CIN2

For peer review only

Table S2. Average number of biopsies by cytology in the electrical impedance spectroscopy (EIS) cohort and in the reference cohort.

	EIS cohort	Reference cohort
	Average number of biopsies	
ASC-US	1.7	2.3
LSIL	1.8	2.2
ASC-H	2.0	2.7
HSIL	2.3	2.8

ASC-US: atypical squamous cells of undetermined significance; LSIL: low-grade squamous intraepithelial lesion; HSIL: high-grade squamous intraepithelial lesion; ASC-H: atypical squamous cells that cannot exclude HSIL

For peer review only

Table S3. Sensitivity, specificity, negative and positive predictive value of the electrical impedance spectroscopy (EIS) cohort and the reference cohort for the detection of CIN2+ lesions by cervical cytology, TZ type and age group.

	EIS cohort (n=647)									Reference cohort (n=962)								
	CIN2+/n	CIN2%	Colpo+ZS CIN2+[1]	Sensitivity	<CIN2/n	Colpo +ZS <CIN2[3]	Specificity	PPV	NPV	CIN2+/n	CIN2%	Colpo CIN2+[2]	Sensitivity	<CIN2/n	Colpo <CIN2[4]	Specificity	PPV	NPV
ASC-US	16/94	17.0	15	94(70-100)	78/94	37	47(36-59)	27(16-40)	97(86-100)	9/99	9.1	5	56(21-86)	90/99	87	97(91-99)	63(25-92)	96(89-99)
LSIL	39/236	16.5	30	77(61-89)	197/236	82	42(35-49)	21(14-28)	90(82-95)	72/381	18.9	31	43(31-55)	309/381	285	92(89-95)	56(42-70)	87(83-91)
ASC-H	85/192	44.3	84	99(94-100)	107/192	12	11(6-19)	47(39-55)	92(64-100)	138/237	58.2	87	63(54-71)	99/237	64	65(54-74)	71(62-79)	56(46-65)
HSIL	77/94	81.9	77	100(95-100)	17/94	1	6(0-29)	83(74-90)	100(3-100)	154/200	77.0	133	86(80-91)	46/200	21	46(31-61)	84(78-90)	50(34-66)
AGC-NOS	3/28	10.7	2	67(9-99)	25/28	11	44(24-65)	13(2-38)	92(62-100)	5/28	17.9	3	60(15-95)	23/28	22	96(78-100)	75(19-99)	92(73-99)
AGC-FN	2/3	66.7	1	50(1-99)	1/3	1	100(3-100)	100(3-100)	50(1-99)	13/17	76.5	8	62(32-86)	4/17	1	25(1-81)	73(39-94)	17(0-64)
ALL	222/647	34.3	209	94(90-97)	425/647	144	34(29-39)	43(38-47)	92(86-96)	391/962	40.6	267	68(63-73)	571/962	480	84(81-87)	75(70-79)	80(76-83)
TZ1	156/446	35.0	146	94(89-97)	290/446	90	31(26-37)	42(37-48)	90(82-95)	279/620	45.0	187	67(61-73)	341/620	287	84(80-88)	78(72-83)	76(71-80)
TZ2	66/201	32.8	63	96(87-99)	135/201	54	40(32-49)	44(36-52)	95(85-99)	112/342	32.7	80	71(62-80)	230/342	193	84(79-88)	68(59-77)	86(81-90)
<30 y	60/175	34.3	56	93(84-98)	115/175	40	35(26-44)	43(34-52)	91(78-98)	134/295	45.4	96	72(63-79)	161/295	124	77(70-83)	72(64-80)	77(69-83)
30-44 y	131/366	35.8	124	95(89-98)	235/366	78	33(27-40)	44(38-50)	92(84-97)	211/495	42.6	144	68(62-75)	284/495	244	86(81-90)	78(72-84)	79(74-83)
>45 y	31/106	29.2	29	94(79-99)	75/106	26	35(24-47)	37(27-49)	93(77-99)	46/172	26.7	27	59(43-73)	126/172	112	89(82-94)	66(49-80)	86(78-91)
HG cytology	164/289	56.7	162	99(96-100)	125/289	14	11(6-18)	59(53-65)	88(62-98)	305/454	67.2	228	75(70-80)	149/454	86	58(49-66)	78(73-83)	53(45-61)
LG cytology	58/358	16.2	47	81(69-90)	300/358	130	43(38-49)	22(16-28)	92(87-96)	86/508	16.9	39	45(35-57)	422/508	394	93(91-96)	58(46-70)	89(86-92)
ASC-H, HSIL	162/286	56.6	161	99(97-100)	124/286	13	11(6-17)	59(53-65)	93(66-100)	292/437	66.8	220	75(70-80)	145/437	85	59(50-67)	79(73-83)	54(46-62)
ASC-US,LSIL	55/330	16.7	45	82(69-91)	275/330	119	43(37-49)	22(17-29)	92(86-96)	81/480	16.9	36	44(33-56)	399/480	372	93(90-96)	57(44-70)	89(86-92)
Glandular	5/31	16.1	3	60(15-95)	26/31	12	46(27-67)	18(4-43)	86(57-98)	18/45	40.0	11	61(36-83)	27/45	23	85(66-96)	73(45-92)	77(58-90)
1 biopsy	11/165	6.7	7	64(31-89)	154/165	78	51(43-59)	8(4-17)	95(88-99)	14/109	12.8	5	36(13-65)	95/109	94	99(94-100)	83(36-100)	91(84-96)
2 biopsies	78/263	29.7	70	90(81-96)	185/263	43	23(17-30)	33(27-40)	84(71-93)	112/420	26.7	66	59(49-68)	308/420	276	90(86-93)	67(57-77)	86(81-89)
≥3 biopsies	43/84	51.2	43	100	41/84	0	0	51	0	168/305	55.1	113	67(60-74)	137/305	92	67(59-75)	72(64-78)	63(54-70)
LLETZ	90/113	79.6	89	99(94-100)	23/113	1	4(0-22)	80(72-87)	50(1-99)	97/118	82.2	83	86(77-92)	21/118	8	38(18-62)	87(78-93)	36(17-59)

EIS: electrical impedance spectroscopy; CIN: cervical intraepithelial neoplasia; TZ: transformation zone; PPV: positive predictive value; NPV: negative predictive value; ASC-US: atypical squamous cells of undetermined significance; LSIL: low-grade squamous intraepithelial lesion; HSIL: high-grade squamous intraepithelial lesion; ASC-H: atypical squamous cells that cannot exclude HSIL; AGC-NOS: atypical glandular cells not otherwise specified; AGC-FN: atypical glandular cells that favor neoplasia; LLETZ: large loop excision of the transformation zone; HG: high grade; LG: low grade

[1]Colposcopic impression and/or ZedScan result of CIN2+ of histologically confirmed CIN2+ cases.

[2]Colposcopic impression of CIN2+ of histologically confirmed CIN2+ cases.

[3]Colposcopic impression and ZedScan result less than CIN2 of histologically confirmed cases <CIN2.

[4]Colposcopic impression less than CIN2 of histologically confirmed cases <CIN2

For peer review only

Table S4. Sensitivity and specificity of the electrical impedance spectroscopy cohort (EIS) and the reference cohort by cytology, TZ type and age group in identifying CIN2+, with corresponding risk differences and the risk ratios of sensitivity and specificity.

	EIS	Reference	Sensitivity			EIS	Reference	Specificity		
	Sensitivity	Sensitivity	Risk difference (95%)[1]	RR (95%)[1]	p.	Specificity	Specificity	Risk difference (95%)[1]	RR (95%)[1]	p.
ASC-US	94(70-100)	56(21-86)	0.38(0.04-0.73)	1.69(0.93-3.07)	0.0219	47(36-59)	97(91-99)	-0.49(-0.61--0.38)	0.49(0.39-0.62)	<0.0001
LSIL	77(61-89)	43(31-55)	0.34(0.16-0.51)	1.79(1.30-2.45)	0.0006	42(35-49)	92(89-95)	-0.51(-0.58--0.43)	0.45(0.38-0.53)	<0.0001
ASC-H	99(94-100)	63(54-71)	0.36(0.27-0.44)	1.57(1.38-1.78)	<0.0001	11(6-19)	65(54-74)	-0.53(-0.65--0.42)	0.17(0.10-0.30)	<0.0001
HSIL	100(95-100)	86(80-91)	0.14(0.08-0.19)	1.16(1.09-1.23)	0.0007	6(0-29)	46(31-61)	-0.40(-0.58--0.22)	0.13(0.02-0.89)	0.0033
AGC-NOS	67(9-99)	60(15-95)	0.07(-0.62-0.75)	1.11(0.38-3.25)	0.8504	44(24-65)	96(78-100)	-0.52(-0.73--0.30)	0.46(0.29-0.72)	0.0001
AGC-FN	50(1-99)	62(32-86)	-0.12(-0.86-0.63)	0.81(0.19-3.47)	0.7565	100(3-100)	25(1-81)	0.75(0.33-1.17)	4.0(0.73-21.84)	0.1709
ALL	94(90-97)	68(63-73)	0.26(0.20-0.31)	1.38(1.28-1.49)	<0.0001	34(29-39)	84(81-87)	-0.50(-0.56--0.45)	0.40(0.35-0.46)	<0.0001
TZ1	94(89-97)	67(61-73)	0.27(0.20-0.33)	1.40(1.27-1.53)	<0.0001	31(26-37)	84(80-88)	-0.53(-0.60--0.47)	0.37(0.31-0.44)	<0.0001
TZ2	96(87-99)	71(62-80)	0.24(0.14-0.34)	1.34(1.18-1.57)	0.0001	40(32-49)	84(79-88)	-0.44(-0.53--0.34)	0.48(0.38-0.59)	<0.0001
<30 y	93(84-98)	72(63-79)	0.22(0.12-0.32)	1.30(1.15-1.48)	0.0007	35(26-44)	77(70-83)	-0.42(-0.53--0.31)	0.45(0.35-0.59)	<0.0001
30-44 y	95(89-98)	68(62-75)	0.26(0.19-0.34)	1.39(1.25-1.53)	<0.0001	33(27-40)	86(81-90)	-0.53(-0.60--0.45)	0.39(0.32-0.47)	<0.0001
≥45 y	94(79-99)	59(43-73)	0.35(0.18-0.52)	1.59(1.23-2.07)	0.0008	35(24-47)	89(82-94)	-0.54(-0.66--0.42)	0.39(0.28-0.54)	<0.0001
HG cytology	99(96-100)	75(70-80)	0.24(0.19-0.29)	1.32(1.24-1.41)	<0.0001	11(6-18)	58(49-66)	-0.47(-0.56--0.37)	0.19(0.12-0.32)	<0.0001
LG cytology	81(69-90)	45(35-57)	0.36(0.21-0.50)	1.79(1.37-2.33)	<0.0001	43(38-49)	93(91-96)	-0.50(-0.56--0.44)	0.46(0.41-0.53)	<0.0001
1 biopsy	64(31-89)	36(13-65)	0.28(-0.10-0.66)	1.78(0.77-4.10)	0.1654	51(43-59)	99(94-100)	-0.48(-0.56--0.40)	0.51(0.44-0.60)	<0.0001
2 biopsies	90(81-96)	59(49-68)	0.31(0.19-0.42)	1.52(1.28-1.81)	<0.0001	23(17-30)	90(86-93)	-0.66(-0.73--0.59)	0.26(0.20-0.34)	<0.0001
≥3 biopsies	100	67(60-74)	0.33(0.26-0.40)	1.49(1.34-1.65)	<0.0001	0	67(59-75)	-0.67(-0.75--0.59)	0	<0.0001

EIS: electrical impedance spectroscopy; CIN: cervical intraepithelial neoplasia; TZ: transformation zone; ASC-US: atypical squamous cells of undetermined significance; LSIL: low-grade squamous intraepithelial lesion; HSIL: high-grade squamous intraepithelial lesion; ASC-H: atypical squamous cells that cannot exclude HSIL; AGC-NOS: atypical glandular cells not otherwise specified; AGC-FN: atypical glandular cells that favor neoplasia; HG: high grade; LG: low grade

[1]The values of risk difference >0 or the values of risk ratio >1 imply better/improved effect with ZedScan.

For peer review only

Section & Topic	No	Item	Reported on page #
**TITLE OR ABSTRACT**			
	**1**	Identification as a study of diagnostic accuracy using at least one measure of accuracy (such as sensitivity, specificity, predictive values, or AUC)	2
**ABSTRACT**			
	**2**	Structured summary of study design, methods, results, and conclusions (for specific guidance, see STARD for Abstracts)	2
**INTRODUCTION**			
	**3**	Scientific and clinical background, including the intended use and clinical role of the index test	4-5
	**4**	Study objectives and hypotheses	4-5
**METHODS**			
Study design	**5**	Whether data collection was planned before the index test and reference standard were performed (prospective study) or after (retrospective study)	5-6
Participants	**6**	Eligibility criteria	5-6
	**7**	On what basis potentially eligible participants were identified (such as symptoms, results from previous tests, inclusion in registry)	5-6
	**8**	Where and when potentially eligible participants were identified (setting, location and dates)	5-6
	**9**	Whether participants formed a consecutive, random or convenience series	5-6
Test methods	**10a**	Index test, in sufficient detail to allow replication	6
	**10b**	Reference standard, in sufficient detail to allow replication	6
	**11**	Rationale for choosing the reference standard (if alternatives exist)	5-6
	**12a**	Definition of and rationale for test positivity cut-offs or result categories of the index test, distinguishing pre-specified from exploratory	7
	**12b**	Definition of and rationale for test positivity cut-offs or result categories of the reference standard, distinguishing pre-specified from exploratory	7
	**13a**	Whether clinical information and reference standard results were available to the performers/readers of the index test	6
	**13b**	Whether clinical information and index test results were available to the assessors of the reference standard	5-6
Analysis	**14**	Methods for estimating or comparing measures of diagnostic accuracy	7
	**15**	How indeterminate index test or reference standard results were handled	5-6
	**16**	How missing data on the index test and reference standard were handled	5-6
	**17**	Any analyses of variability in diagnostic accuracy, distinguishing pre-specified from exploratory	7
	**18**	Intended sample size and how it was determined	5-6
**RESULTS**			
Participants	**19**	Flow of participants, using a diagram	Figure S1, submitted as a separate file
	**20**	Baseline demographic and clinical characteristics of participants	9, Table 1
	**21a**	Distribution of severity of disease in those with the target condition	9, Table 1
	**21b**	Distribution of alternative diagnoses in those without the target condition	9, Table 1
	**22**	Time interval and any clinical interventions between index test and reference standard	5-6
Test results	**23**	Cross tabulation of the index test results (or their distribution) by the results of the reference standard	9,10,11 Table1-3, Table S3-4
	**24**	Estimates of diagnostic accuracy and their precision (such as 95% confidence intervals)	10,11 Table 2-3, Table S3-4
	**25**	Any adverse events from performing the index test or the reference standard	NA
**DISCUSSION**			
	**26**	Study limitations, including sources of potential bias, statistical uncertainty, and generalisability	13-16
	**27**	Implications for practice, including the intended use and clinical role of the index test	15-16
**OTHER INFORMATION**			

For peer review only

CC BY SA

	**28**	Registration number and name of registry	6 ISRCTN for the reference cohort
	**29**	Where the full study protocol can be accessed	Submitted as a separate file
	**30**	Sources of funding and other support; role of funders	16

For peer review only

STARD 2015

AIM

STARD stands for "Standards for Reporting Diagnostic accuracy studies". This list of items was developed to contribute to the completeness and transparency of reporting of diagnostic accuracy studies. Authors can use the list to write informative study reports. Editors and peer-reviewers can use it to evaluate whether the information has been included in manuscripts submitted for publication.

EXPLANATION

A **diagnostic accuracy study** evaluates the ability of one or more medical tests to correctly classify study participants as having a **target condition.** This can be a disease, a disease stage, response or benefit from therapy, or an event or condition in the future. A medical test can be an imaging procedure, a laboratory test, elements from history and physical examination, a combination of these, or any other method for collecting information about the current health status of a patient.

The test whose accuracy is evaluated is called **index test.** A study can evaluate the accuracy of one or more index tests. Evaluating the ability of a medical test to correctly classify patients is typically done by comparing the distribution of the index test results with those of the **reference standard**. The reference standard is the best available method for establishing the presence or absence of the target condition. An accuracy study can rely on one or more reference standards.

If test results are categorized as either positive or negative, the cross tabulation of the index test results against those of the reference standard can be used to estimate the **sensitivity** of the index test (the proportion of participants *with* the target condition who have a positive index test), and its **specificity** (the proportion *without* the target condition who have a negative index test). From this cross tabulation (sometimes referred to as the contingency or "2x2" table), several other accuracy statistics can be estimated, such as the positive and negative **predictive values** of the test. Confidence intervals around estimates of accuracy can then be calculated to quantify the statistical **precision** of the measurements.

If the index test results can take more than two values, categorization of test results as positive or negative requires a **test positivity cut-off**. When multiple such cut-offs can be defined, authors can report a receiver operating characteristic (ROC) curve which graphically represents the combination of sensitivity and specificity for each possible test positivity cut-off. The **area under the ROC curve** informs in a single numerical value about the overall diagnostic accuracy of the index test.

The **intended use** of a medical test can be diagnosis, screening, staging, monitoring, surveillance, prediction or prognosis. The **clinical role** of a test explains its position relative to existing tests in the clinical pathway. A replacement test, for example, replaces an existing test. A triage test is used before an existing test; an add-on test is used after an existing test.

Besides diagnostic accuracy, several other outcomes and statistics may be relevant in the evaluation of medical tests. Medical tests can also be used to classify patients for purposes other than diagnosis, such as staging or prognosis. The STARD list was not explicitly developed for these other outcomes, statistics, and study types, although most STARD items would still apply.

DEVELOPMENT

This STARD list was released in 2015. The 30 items were identified by an international expert group of methodologists, researchers, and editors. The guiding principle in the development of STARD was to select items that, when reported, would help readers to judge the potential for bias in the study, to appraise the applicability of the study findings and the validity of conclusions and recommendations. The list represents an update of the first version, which was published in 2003.

More information can be found on http://www.equator-network.org/reporting-guidelines/stard.

CC BY SA
